# Mechanistic insight into the impact of nanomaterials on asthma and allergic airway disease

**DOI:** 10.1186/s12989-017-0228-y

**Published:** 2017-11-21

**Authors:** Kirsty Meldrum, Chang Guo, Emma L. Marczylo, Timothy W. Gant, Rachel Smith, Martin O. Leonard

**Affiliations:** Toxicology Department, Centre for Radiation, Chemical and Environmental Hazards, Public Health England, Chilton, Harwell Campus, OX11 0RQ UK

**Keywords:** Asthma, Allergy, Nanomaterials, Nanoparticles, Ultrafine Pollutant, Inhalation, Lung

## Abstract

Asthma is a chronic respiratory disease known for its high susceptibility to environmental exposure. Inadvertent inhalation of engineered or incidental nanomaterials is a concern for human health, particularly for those with underlying disease susceptibility. In this review we provide a comprehensive analysis of those studies focussed on safety assessment of different nanomaterials and their unique characteristics on asthma and allergic airway disease. These include *in vivo* and *in vitro* approaches as well as human and population studies. The weight of evidence presented supports a modifying role for nanomaterial exposure on established asthma as well as the development of the condition. Due to the variability in modelling approaches, nanomaterial characterisation and endpoints used for assessment in these studies, there is insufficient information for how one may assign relative hazard potential to individual nanoscale properties. New developments including the adoption of standardised models and focussed *in vitro* and *in silico* approaches have the potential to more reliably identify properties of concern through comparative analysis across robust and select testing systems. Importantly, key to refinement and choice of the most appropriate testing systems is a more complete understanding of how these materials may influence disease at the cellular and molecular level. Detailed mechanistic insight also brings with it opportunities to build important population and exposure susceptibilities into models. Ultimately, such approaches have the potential to more clearly extrapolate relevant toxicological information, which can be used to improve nanomaterial safety assessment for human disease susceptibility.

**Electronic supplementary material:**

The online version of this article (10.1186/s12989-017-0228-y) contains supplementary material, which is available to authorized users.

## Background

Asthma affects over 330 million individuals worldwide and is associated with significant avoidable mortality, morbidity and economic burden [1]. In pathophysiological terms, asthma is a chronic inflammatory condition of the airway and is increasingly considered a term to cover a broad spectrum disease category with multiple causes and phenotypes [2]. Clinical symptoms include airway obstruction, bronchospasm, wheezing, coughing, shortness of breath and airway hyper-responsiveness (AHR) [3]. The most common underlying disease process is chronic inflammation, which encompasses a wide array of resident and immune cell types. As a consequence of this inflammation, airway remodelling occurs with specific changes manifesting as sub-epithelial fibrosis, smooth muscle thickening, neo-vascularisation and epithelial barrier modification. This restructuring together with increased smooth muscle contractility and enhanced mucus secretion cause obstructive events and the clinical symptoms of disease [4–7].

There are a number of environmental factors with strong links to the development of asthma, including early *in utero* and childhood exposure to microbes, infection, diet, obesity, vitamin D levels, allergens, chemicals and tobacco smoke [8–17]. Exposures, particularly to allergens, particulates and chemicals, and respiratory infections also account for the majority of known triggers for obstructive events in asthma [9, 18–20]. It has been recognised for some time that certain chemicals can cause asthma and allergic airway disease (AAD) [21, 22]. Properties intrinsic to these chemicals and more recently for particulate exposure, have been suggested to govern their ability to produce adverse effects. Nanomaterials (NMs), including ultrafine particulates, defined as having at least one dimension less than 100nm [23, 24], are recognised as possessing not simply chemical but also physical characteristics, with the potential to modify disease risk and outcome.

Concerns have been raised regarding the safety of both engineered NMs and incidental ultrafine particulates upon inadvertent exposure in humans [25]. Epidemiological approaches to understanding how nanoscale materials affect asthma and AAD have been restricted to a handful of studies with limited power to attribute causal effect. The study of pollutant mixtures has suggested that nanoscale components of fossil fuel combustion products such as diesel exhaust particulates may contribute to asthma and allergic airway disease [20, 26, 27]. As chemicals including polycyclic aromatic hydrocarbons and quinones adhered to ultrafine particles are increasingly suggested as the primary factor responsible for adverse effects [26, 28–30], it is important to properly define the nanoscale characteristic contribution. Such information also has the potential to increase our understanding of how engineered nanoscale materials may impact asthma. As a focus for this review, we will describe current knowledge from human and experimental observations, on how respiratory exposure to nanomaterials with different compositions and characteristics modify asthma and AAD. We will also describe current understanding of the mechanisms through which such modification can occur, and explore important knowledge gaps to allow prioritisation of research focus into the future.

There are different ways in which nanomaterial (NM) effects on asthma can be considered. In terms of experimental modelling, there are those approaches that have examined either naïve or genetically susceptible animals in the absence of allergen initiated disease. There are other approaches that have used allergen induced inflammation as a conditioning step for disease initiation and used different protocols to examine effects on the development (adjuvancy) or exacerbation of established allergic airway disease. Using the search criteria detailed in Additional file 1 to encompass all aspects of disease susceptibility, we have performed a comprehensive analysis of those studies, which examine the effect of pulmonary exposure to nanoparticles (NPs), including nano-sized ultrafine pollutants, on humans and experimental models of asthma and AAD. Those studies focussed on medical applications of NMs were excluded from the main analysis and discussed separately.

## Experimental models and evidence for modifying effect of nanomaterials

Experimental modelling as a means to identify hazards and develop risk assessment and management approaches is fundamental to how materials are assessed for potential safety concerns. The majority of such testing is carried out using *in vivo* exposure systems and occasionally incorporates models of disease susceptibility. A number of experimental *in vivo* approaches have been used to assess the impact of NMs on asthma and AAD. In order to interpret these findings as translational information that can be applied to human exposure, we must first describe the pathological features of asthma and its endotype subcategories as measurable endpoints that can be experimentally modelled and assessed.

Current approaches to the categorisation of asthma involve subdivision based on immunological characteristics as well as clinical symptoms and severity of disease [2]. The most common form of asthma found in children and half of adults is atopic in nature and correlates with increased IgE antibody levels and/or a positive skin prick test for allergens such as house dust mite (HDM). Increased IgE levels in atopic conditions are a functional consequence of CD4+ T-cell patterning towards a Th2 phenotype, which switches B-cell antibody production to IgE. In AAD, IgE coated eosinophils, mast cells and basophils are activated upon re-exposure to allergen and an inflammatory response is initiated. Allergen specific activation of Th2 CD4+ T-cells in lung tissue increases the production of IL-4, IL-5 and IL-13 cytokines. This results in an increase in IgE production from B-cells, eosinophil recruitment and goblet cell metaplasia as well as  increased mucus production (MUC5AC), all of which contribute to airway restriction [31]. Within the category of a high Th2 asthma endotype, there is a subdivision of disease, which displays eosinophilia and a Th2 cytokine environment in the absence of an adaptive immune response and is often referred to as intrinsic asthma [32]. It has been proposed that the newly discovered innate lymphoid ILC2 cells drive this type of disease in response to chronic stimulation from infection, pollution and irritants. Injury to and activation of the epithelial cell layer in the airway is a key feature of Th2 mediated responses with epithelial derived factors including IL-33, TSLP, IL-25 and GM-CSF all suggested to play a role [31].

Another important endotype typically associated with late onset and more severe forms of asthma is one where there is a predominant neutrophilic inflammation and the presence of a mixed Th1 and Th17 immune response, producing IL-17A, IL-22, IFNγ and TNFα among other mediators [33]. It is also important to consider overlapping endotypes, where individuals will have varying degrees of inflammation and disease severity, for example combined eosinophilic and neutrophilic disease with a Th2 and Th17 profile [34]. Airway obstruction, hyperresponsiveness, remodelling (cellular proliferation & extracellular matrix deposition) and excessive mucus production with goblet cell hyperplasia are common features across all endotypes of asthma and are directly measureable in model systems [35, 36]. Together with endotype specific endpoints as described above, they can all be used to model the impact of material exposure on disease outcome.

### Nanomaterial exposure and the development of allergic airway disease

Models of atopic asthma typically involve sensitisation to allergens such as ovalbumin (OVA) in rodents and testing of materials for their ability to modify allergic inflammation and airway function, and have allowed a greater understanding of key events in asthmatic disease progression. NM testing for their impact on the sensitisation steps in these models aims to examine how such materials may influence the development and severity of new cases of the disease, which includes adjuvant activity and have been summarized in Table 1. One of the earliest studies investigating adjuvant activity of NPs demonstrated that carbon nanoparticles (CNPs) with a smaller size (14nm) and greater surface area aggravated OVA induced allergic inflammation and mucus hypersecretion, while larger sized particles (56nm) of the same material did not [37]. Similar adjuvant effects were observed in other studies from this group using the same particles [38] [39] but there was not always a size dependent distinction for example when examining AHR [40]. The adjuvant effects of CNPs have also been observed in studies from other groups [41] and display dose dependent effects [42] as well as material composition dependent differences when compared to titanium dioxide NPs (TiO_2_NPs) for example [43].

**Table 1:** Effect of nanomaterials on development of allergic airway disease *in vivo*

Nanomaterial Properties				Model Characteristics				Disease Related Endpoints									
*Material (coating)*	*Size (nm)*	*Surface area (m* ^*2*^ */g)*	*Agg Size: nm*	*Species (Strain - Gender)*	*Allergen (Route)*	*Particle (Route)*	*Dose*	*AHR (LF test)*	*IgE*	*Eos*	*Neu*	*Lym*	*MΦ*	*Total Cells*	*Pathophysiological Indicators*	*GCH-MS*	*Reference*
C	14	300	-	Mice (ICR –m)	OVA (Int T)	Int T	1.61 mg/kg x6	-	↑	↑	↑	-	↑	↑	↑ (IL5, IL13, MCP1, IL6); nc (IL4)	↑ Goblet cells	[37]
C	56	45	-	Mice (ICR –m)	OVA (Int T)	Int T	1.61 mg/kg x6	-	nc	nc	nc	-	nc	↑	↑ (IL5, IL4, IL6, MCP1); nc (IL13)	nc Goblet cells	[37]
C	14	300	-	Mice (ICR –m)	OVA (Int T)	Int T	1.61 mg/kg x6	-	-	-	-	-	-	-	↑ (MIP1α, IL2, IL10, TARC)	-	[38]
C	56	45	-	Mice (ICR –m)	OVA (Int T)	Int T	1.61 mg/kg x6	-	-	-	-	-	-	-	↑ (MIP1α, TARC), nc (IL2, IL10)	-	[38]
C	14	300	-	Mice (ICR –m)	OVA (Int T)	Int T	1.61 mg/kg x6	↑ (Resist)	-	-	-	-	-	-	-	nc MUC5AC	[40]
C	56	45	-	Mice (ICR –m)	OVA (Int T)	Int T	1.61 mg/kg x6	↑ (Resist)	-	-	-	-	-	-	-	↑ MUC5AC	[40]
C	14	-	887	Mice (BALB/c –f)	OVA (PA)	PA	2.5 mg/kg x4	-	↑	↑	↑	-	nc	↑	↑ (CXCL1, IL13), nc (IL4)	-	[41]
C	30-50	-	-	Mice (BALB/c –f)	OVA (IP)	Int N	11 mg/kg	-	↑	↑	nc	nc	nc	↑	↑ TNFα, IL4, IL5, IL10, INFγ	↑ PAS	[42]
C	14.3	253.9	-	Mice (BALB/c –f)	OVA (Int N)	Int N	3.6 mg/kg x3	-	nc	↑	↑	nc	nc	↑	↑ (IL4, IL5, IL10, IL13, INFγ, TNFα)	-	[43]
C	30-50	-	-	Mice (BALB/c –f)	OVA (Int N)	Int N	3.6 mg/kg x3	-	-	-	-	-	nc	-	↑ (IL-4, IL-5, IL-10, IL-13, IFN-γ, TNFα)	-	[72]
C	14	300	-	Mice (ICR –m)	OVA (Int T)	Int T	1.61 mg/kg x6	-	-	-	-	-	↑	↑	↑ (CD86, CD80, MHC II, DEC205, CD19)	-	[39]
C	56	45	-	Mice (ICR –m)	OVA (Int T)	Int T	1.61 mg/kg x6	-	-	-	-	-	nc	nc	nc (CD86, CD80, MHC II, DEC205, CD19)	-	[39]
C	13	-	<10 μm	Rat (BN –m)	OVA (IP)	Int T	0.5 mg/kg x3	nc (Resist)	nc	↓	nc	↑	nc	nc	nc (INFγ, IL1β, TNFα, IL4, IL5, IL13)	-	[238]
TiO_2_	29	49.8	-	Mice (BALB/c –f)	OVA (Int N)	Int N	11 mg/kg	-	↑	nc	nc	nc	nc	↑	↑ (TNFα, IL4, IL5, IL10, IL13) nc(INF-γ)	-	[43]
C_70_ Fullerene	-	-	-	Mice (BALB/c)	OVA (IP)	Int N	1.1 mg/kg	↓ (Resist)	↓	↓	nc	-	nc	↓	↓ (IL4, IL5), ↑ (CYP1B1)	-	[106]
Fe_2_O_3_	35	39	-	Mice (BALB/c –f)	OVA (IP)	Int T	27.7 mg/kg x4	-	↓	↓	nc	↓	-	-	↓ IL4, INFγ	-	[80]
GO	D 0.6 L 0.02-2μm	-	-	Mice (BALB/c –f)	OVA (IP)	PA	2.2 mg/kg	↑ (Penh)	↓	↓	nc	nc	↑	-	↓ (IL4, IL13) nc (IL5)	↑ GCH/PAS	[65]
Latex	25	-	-	Mice (ICR –m)	OVA (Int T)	Int T	13.8 mg/kg	-	nc	nc	↑	↑	↑	↑	↑ (IL18, MIP1α, MCP1); nc (IL5, IL13)	-	[272]
Latex	50	-	-	Mice (ICR –m)	OVA (Int T)	Int T	13.8 mg/kg	-	nc	nc	↑	↑	↑	↑	↑ (IL18, MIP1α, MCP1); nc (IL5, IL13)	-	[272]
Latex	100	-	-	Mice (ICR –m)	OVA (Int T)	Int T	13.8 mg/kg	-	nc	nc	nc	nc	nc	nc	↑ (IL18, MIP1α, MCP1); nc (IL5, IL13)	-	[272]
PS (Glycine)	50	-	-	Mice (BALB/c –f)	OVA (IP)	Int T	11 mg/kg	-	-	↓	nc	↓	nc	↓	↓ (IL5, IL13)	-	[79]
PS	59	-	-	Mice (BALB/c –f)	OVA (Int N)	Int N	16 mg/kg x3	-	↑	↑	↑	↑	↑	↑	-	-	[44]
PS	59	-	-	Mice (BALB/c –m)	OVA (Int N)	Int N	16 mg/kg x3	-	↑	↑	↑	↑	↑	↑	-	-	[44]
PS (Glycine)	45-49	-	-	Mice (BALB/c)	OVA (Int T)	Int T	11 mg/kg x2	nc	↓	↓	nc	nc	nc	↓	↓ (IL-4, IL-5, IL-13), nc (IFN-γ)	↓ PAS	[78]
SiO_2_	7	300	-	Mice (C57BL/6-f)	OVA (Inh)	PA	2.7 mg/kg	-	nc	-	-	-	-	-	↑(HO1); nc(TNFα,,IL1β, Cxcl2, Ccl2)	-	[55]
SiO_2_	34	80	-	Mice (C57BL/6-f)	OVA (Inh)	PA	2.7 mg/kg	-	nc	-	-	-	-	-	↑(HO1); nc(TNFα,,IL1β, Cxcl2, Ccl2)	-	[55]
TiO_2_ (Al(OH)_3_)	30-50	37.1	-	Mice (C57BL/6-f)	OVA (Inh)	PA	2.7 mg/kg	-	nc	-	-	-	-	-	nc (HO1, TNFα, IL1β, Cxcl2, Ccl2)	-	[55]
ZnO	21	49.6	-	Mice (C57BL/6-f)	OVA (Inh)	PA	2.7 mg/kg	-	↑	-	-	-	-	-	nc (HO1, TNFα, IL1β, Cxcl2, Ccl2)	-	[55]
ZnO (SiO_2_)	25	-	-	Mice (C57BL/6-f)	OVA (Inh)	PA	2.7 mg/kg	-	↑	-	-	-	-	-	-	-	[55]
SiO_2_	10-20	140-180	-	Rat (Wistar –m)	OVA (IP)	Int T	1.6 mg/kg x30	↑ (Resist)	-	↓	-	-	-	-	↑ (IL-4), nc (INF-γ)	-	[46]
SiO_2_ (PEG)	90	-	-	Mice (BALB/c –f)	OVA (Int N)	Int N	22.2 mg/Kg x4	-	↑	↑	↑	↑	nc	↑	↑ (IL-6, IL-5, IL-1β, IL-4, TNF-α, INF-γ	↑ PAS, MUC5AC	[47]
SiO_2_	100	12.7	119.6	Mice (BALB/c –f)	OVA (Int N)	Int N	10 mg/kg x6	↑ (Resist)	-	↑	↑	↑	nc	↑	↑ (IL-5, IL-13, IL-1β, INF-γ	nc PAS	[48]
SiO_2_ (Meso)	100	70.6	100.5	Mice (BALB/c –f)	OVA (Int N)	Int N	10 mg/kg x6	↑ (Resist)	-	↑	↑	↑	↑	↑	↑ (IL-1β, INF-γ) nc (IL-5, IL-13)	↑ PAS	[48]
SiO_2_ (PEG)	100	12.7	439.1	Mice (BALB/c –f)	OVA (Int N)	Int N	10 mg/kg x6	nc (Resist)	-	nc	nc	nc	nc	nc	↑ (INF-γ) nc (IL-1β, IL-5, IL-13)	nc PAS	[48]
TiO_2_	21	-	1.5 μm	Mice (BALB/c –f)	OVA (IP)	A (Nose only)	193μg, deposited	nc (resist)	↓	↓	nc	↓	nc	↓	↓ (IL-5, IL-6, INF-γ, IL-13, IL-4)	-	[120]
TiO_2_ (SiO_2_)	10x40	132	100	Mice (BALB/c –f)	OVA (IP)	A	10mg/m^3^ 2h/d x12d	↓ (Penh)	nc	↓	nc	↓	↓	-	↓ (IL-1β, TNF-α, IL-4, IL-13, IL-10)	↓ PAS	[81]
TiO_2_	4-8	130	244	Mice (BALB/c)	OVA (IP)	Int N	11 mg/kg	↑ (Resist)	↑	-	-	-	-	-	nc (IL-5) ↑ (IL-4, IL-13,IL-6, TNFa)	-	[273]
ZnO	50	10.8	-	Mice (Balb/c –f)	OVA (OA)	OA	0.5 mg/kg x2	-	nc	↑	-	-	-	↑	↑ (IL-4, IL-5, IL-6, IL-13)	-	[56]
CNF	D-35, L-10μm	103	-	Mice (BALB/c –f)	OVA (IP)	Int N	7.4mg/kg x3	-	↑	nc	nc	nc	nc	nc	nc (MCP-1, TNF-α)	-	[54]
CNF	D- 36, L-5μm	124	-	Mice (BALB/c –f)	OVA (IP)	Int N	7.4mg/kg x3	-	↑	nc	↑	nc	↑	↑	nc (MCP-1, TNF-α)	-	[54]
CNF	D-70, L-5μm	56	-	Mice (BALB/c –f)	OVA (IP)	Int N	7.4mg/kg x3	-	↑	nc	nc	nc	nc	nc	nc (MCP-1, TNF-α)	-	[54]
CNF	D- 83, L-10μm	61	-	Mice (BALB/c –f)	OVA (IP)	Int N	7.4mg/kg x3	-	↑	nc	↑	nc	nc	↑	nc (MCP-1, TNF-α)	-	[54]
SWCNT	D- 1.1, L-100μm	543	-	Mice (BALB/c –f)	OVA (IP)	Int N	7.4mg/kg x3	-	↑	↑	↑	↑	↑	↑	nc (MCP-1, TNF-α)	-	[54]
MWCNT	D20-30, L-200μm	140	-	Mice (BALB/c –f)	OVA (IP)	Int N	7.4mg/kg x3	-	↑	↑	↑	nc	↑	↑	nc (MCP-1, TNF-α)	-	[54]
C (PrinteX 90)	14	300		Mice (BALB/c –f)	OVA (IP)	Int N	7.4mg/kg x3	-	↑	↑	↑	↑	↑	↑	↑(MCP-1), nc (TNF-α)	-	[54]
SWCNT	D 1.2-2, L 1-15μm	-	-	Mice (ICR –m)	OVA (Int T)	Int T	1.3 mg/kg x7	-	↑	↑	↑	↑	↑	↑	↑ (IL4, IL33, IL5, IL13, INFγ, IL17)	↑ MUC5AC	[52]
SWCNT	D 0.8-1.2, L 0.1-1μm	-	-	Mice (ICR –m)	OVA (Int T)	Int T	1.3 mg/kg x7	-	↑	↑	↑	↑	nc	↑	↑ (IL5, IL13)	↑ MUC5AC	[52]
MWCNT	D ~15 nm, L 1-10μm	-	-	Mice (BALB/c –m)	OVA (Int N)	Int N	1mg/kg	↑ (sRaw)	↑	↑	↑	↑	↑	↑	↑ (IL-4, IL-5, IL-13, IL-17)	-	[50]
SWCNT	D 0.93-1.63	-	-	Rat (Wistar –m)	OVA (Int T)	Int T	2 mg/kg x13	↑ (Resist)	↑	↑	↑	↑	-	↑	↑ (IL4, ROS) ↓(INFγ)	↑ PAS	[53]
MWCNT	D ~15 nm, L 1-10μm	-	~350 μm	Mice (BALB/c –m)	HDM (Int N)	Int N	12.5mg/kg x10	-	-	↑	↑	↑	↑	↑	↑ IL-13, TGF-β1, IL-33, TSLP, IL-25	↑ mucin	[51]
MWCNT	D 67, L 0.1-10μm	26	-	Mice (ICR –m)	OVA (Int I)	Int T	4.6 mg/kg x6	-	↑	↑	↑	↑	nc	↑	↑ (IL-4, INF-γ, IL-33, IL-1β, TARC, MCP-1)	↑ PAS	[49]
MWCNT	D 2–20, L 0.1-10μm	-	-	Mice (ICR –m)	OVA (Int I)	Int T	4.6 mg/kg x6	-	↑	↑	↑	↑	nc	↑	↑ (IL-4, INF-γ, IL-33, MCP-1, IL-1β);	-	[49]
SiO_2_	33	-	-	Mice (BALB/c –f)	OVA (Int N)	Int N	13.8 mg/kg x3	-	↑	-	-	-	-	-	↑ (IL-4, IL-5)	-	[45]
SiO_2_	79	-	-	Mice (BALB/c –f)	OVA (Int N)	Int N	13.8 mg/kg x3	-	nc	-	-	-	-	-	nc (IL-4, IL-5)	-	[45]

Abbreviations: *Agg* agglomerate, *AHR* airway hyperresponsiveness, *LF* lung function, *Resist* Resistance, *Penh* Enhanced pause, *Eos* eosinophils, *Neu* neutrophils, *Lym* lymphocytes, MΦ macrophages, *GCH-MS* goblet cell hyperplasia-mucus secretion, −*m* –male, *OVA* ovalbumin, *Int T* intratracheal instillation, ↑ increase, *nc* no change, − not determined, −*f* –female, *PA* pharyngeal aspiration, *Int N* intranasal instillation, *PAS* Periodic acid–Schiff staining, *BN* Brown Norway, *IP* intraperitoneal injection, ↓ decrease, *PS* polystyrene, *Inh* inhalation, *PEG* Polyethylene glycol, *Meso* mesoporous, *OA* oropharyngeal aspiration, *A* aerosol, *D* diameter; *L* length; *CNF* carbon nanofiber, *SWCNT* single-walled carbon nanotubes, *MWCNT* multi-walled carbon nanotubes, *HDM* house dust mite

Comparison to non-nano sized particles has also been carried out within these adjuvant studies and further supports the hypothesis that smaller size and greater surface area have a larger impact on biological reactivity and AAD. This was observed for example, when comparing nano-sized carbon and TiO_2_ to sub-micron sized particles of the same material [43]. It has also been observed with polystyrene particles (PSP), where nano-sized PSP produced stronger allergic inflammatory responses to OVA than sub-micron or micron sized PSPs [44]. Interestingly this study addressed potential gender response differences and observed that while PSPs had adjuvant effects in both male and female mice, there were significantly higher IgE and eosinophilic responses in females when compared to males. Examination of silicon dioxide NPs (SiO_2_NPs) also demonstrated size and dose dependent effects, where 30nm sized SiO_2_NPs increased OVA specific IgE and Th2 type cytokine production while larger sized SiO_2_NPs (70nm) or sub-micron and micron sized silica did not [45]. The adjuvant effects of SiO_2_NPs have been well documented with additional studies revealing dose dependent increases in AHR, allergic as well as innate airway inflammation (both Th2 and Th17 related) and mucus hypersecretion [46, 47]. A more detailed exploration of how SiO_2_NPs have adjuvant effects was recently carried out using three different types of SiO_2_NPs (unmodified, mesoporous and polyethylene glycol (PEG) conjugated). The strongest adjuvant activity, measured as increased AHR, inflammatory responses and mucus hypersecretion, was found for the mesoporous form of SiO_2_NPs. This form has a similar size to the unmodified SiO_2_NPs but has a much larger surface area. It was argued that surface area is the key component for adjuvant activity. This was further supported by the observation that the PEG-SiO_2_NPs, which had agglomerates four times larger, and thus a lower surface area per mass dose, failed to produce any adjuvant activity [48].

Multi-walled carbon nanotubes (MWCNTs) are a particular concern for human health and have been observed to worsen the development of AAI with increased IgE, eosinophils, lymphocytes, IL-4 and mucus hypersecretion in a mouse model of AAD [49]. This was also accompanied by innate immune responses including increased neutrophils, IL-1β and IL-33 [49]. Similar effects for MWCNTs [50, 51] and single walled carbon nanotubes (SWCNTs) [52, 53] were observed in subsequent studies. Intriguingly, an analysis of different carbon NMs revealed that both SWCNTs and MWCNTs produced significantly higher levels of IgE and pulmonary eosinophils than either carbon nanofibres (CNFs) or CNPs. CNFs in general had a lesser impact on inflammatory responses and were correlated to a lower specific surface area. Different surface areas however were not sufficient to fully explain allergic airway responses, and nanoparticle (NP) characteristics such as thin tubular structure and biopersistance were suggested as more likely factors contributing to adverse effects [54].

In addition to size, material type and structure, solubility of NPs has also been proposed as an important factor for the ability to modulate the development of AAD. Comparison of different NPs for their potential to modify IgE responses to OVA in mice, revealed that zinc oxide nanoparticles (ZnONPs) but not SiO_2_NPs or TiO_2_NPs produced an adjuvant effect [55]. It was suggested that the higher solubility of ZnONPs as a contributing factor. Similar to effects independent of allergen, worsening of OVA induced allergic airway inflammation has also been demonstrated in response to ZnONPs but not ZnCl_2_ ion treatment [56]. Given the suggestion that the nanoparticle intracellular release of Zn ions over a sustained period of time is the key factor for biological effect [57], the use of an ionic metal bolus may not be entirely appropriate to replicate all effects.

### Nanomaterial effects on established allergic airway disease

Modification of pre-existing asthma involves alterations to airway function and inflammatory status. In this section we will summarise current information on how nanomaterials can modify established allergic airway disease *in vivo* (Table 2).

**Table 2:** Effect of nanomaterials on experimental *in vivo* models of established allergic airway disease

Nanomaterial Properties				Model Characteristics				Disease Related Endpoints									
*Material (coating)*	*Size (nm)*	*Surface area (m* ^*2*^ */g)*	*Agg size (nm)*	*Species (Strain - Gender)*	*Allergen (Route)*	*Particle (Route)*	*Dose*	*AHR (LF test)*	*IgE*	*Eos*	*Neu*	*Lym*	*MΦ*	*Total Cells*	*Pathophysiological Indicators*	*GCH-MS*	*Reference*
Ag	6	0.11	-	Mice (BALBc –f)	OVA (IP)	A	40mg/kg x5d	↓ (Penh)	-	↓	nc	-	nc	↓	↓ (IL-4 IL-5, IL-13, VEGF)	↓ MUC5AC	[112]
Ag	6	-	-	Mice (C57BL/6 –f)	OVA (IP)	A	40mg/kg x5d	↓ (Penh)	-	↓	nc	↓	nc	↓	↓ (IL-4 IL-5, IL-13, NFkB)	-	[111]
Ag	33	-	-	Mice (BALB/c –f)	OVA (IP)	A	3.3mg/m^3^ x6h/d x7d	-	↑	-	-	-	-	-	-	-	[274]
Ag	33	0.016	-	Mice (BALB/c –f)	OVA (Int N)	A	3.3mg/m^3^ x6h x7d	nc (Penh)	nc	nc	nc	nc	nc	nc	nc (IL-13) -	-	[116]
Au	40	-	-	Mice (BALB/c –m)	TDI (dermal)	OA	0.8 mg/kg	↑ (Resist)	nc	nc	↑	-	↑	↑	↓ (TNF-α, IL-6) nc (MIP-2, MCP-1)	-	[63]
Au	6.3	-	-	Mice (A/J –m)	OVA (IP)	Int N	60μg/Kg x2	↓ (Resist)	-	↓	-	-	-	-	↓ (IL-4, IL-13, CCL11, CCL24)	↓ PAS	[117]
Au	6.3	-	-	Mice (Swiss Webster –m)	OVA (IP)	Int N	60μg/Kg x2	-	-	↓	↓	-	↓	↓	↓ (IL-5, Il-1β, IL-6, ROS)	-	[117]
TiO_2_	22	-	272	Mice (BALB/c –m)	TDI (dermal)	OA	0.8 mg/kg	nc (Resist)	nc	nc	↑	-	↑	↑	nc (TNF-α, IL-6, MIP-2, MCP-1)	-	[63]
CuO	46.67	-	375.8	Mice 0(BALB/c –f)	OVA (IP)	Int N	100μg/kg x3	↑ (Penh)	↑	↑	↑	↑	↑	↑	↑ (ROS, IL-5, IL-6, TNF-α, IL-13, IL-4	↑ MUC5AC	[64]
C	20	442	344-5720	Mice (C57BL/6 –f)	OVA (IP)	PA	10mg/kg	-	-	-	↑	-	nc	↑	-	-	[60]
C	34.8	-	-	Mice (BALB/c)	OVA (A)	A	526μg/m^3^ x24h	↑ (Penh)	-	↑	-	↑	↑	-	↑(IL-13), ↓(IL-5), nc (IL-4)	↑ PAS	[61]
C	53.2	-	-	Mice (BALB/c –f)	OVA (IP)	A	504μg/m3 (24h)	↑ (Penh)	-	↑	-	↑	nc	↑	↑ (IL-5, IL-13)	-	[110]
C	35.2	-	-	Dog (Beagle)	Ragweed (IP)	A	232.3μg/m^3^	nc (Resist)	nc	nc	↑	nc	nc	nc	-	-	[58]
C	7	-	-	Mice (BALB/c –f)	OVA (IP)	A	507μg/m^3^ x24h	-	-	nc	↑*	-	nc	-	↑ (LTB_4_, PGE_2_), nc (8-isoprostane)	-	[59]
C	7	-	-	Mice (BALB/c)	OVA (IP)	A	507μg/m^3^ x24h	-	-	-	-	-	-	-	↑ (TNF-α), nc (CC16)	↑ PAS	[62]
α-Fe_2_O_3_	30	28.98	950-2000	Mice (BALB/c –f)	OVA (IP)	Int T	5mg/kg	-	nc	↓	nc	↓	↓	↓	nc (TNF-α, Il-1β, IL6)	-	[275]
FeO_2_	43	41.95	200	Mice (BALB/c –m)	OVA (IP)	Int T	1.1mg/kg x3	-	-	-	-	↑	-	↑	↑ (IFN-γ), nc (IL-4)	-	[93]
GO	D 0.61, L 0.02-2μm	-	-	Mice (BALB/c –f)	OVA (IP)	PA	2.2mg/kg x4	nc (Penh)	nc	nc	↑	↓	nc	-	nc (IL-4, IL-5, IL-13)	-	[65]
SiO_2_	14	-	-	Mice (BALB/c –f)	OVA (IP)	Int T	2.7mg/kg	nc (Resist)	-	↑	↑	↑	↓	-	↑ (IL-13, MCP-1, MIP-1, Tarc); nc (IL-4, INF-γ, TNF-α, KC)	↑ MUC5AC PAS	[66]
SiO_2_ (PEG)	14	-	-	Mice (BALB/c –f)	OVA (IP)	Int T	2.7mg/kg	↑ (Resist)	-	↑	↑	↑	↓	-	↑ (IL-4, IL-13, MCP-1, MIP-1, Tarc, KC), nc (INF-γ, TNF-α)	↑ MUC5AC PAS	[66]
SiO_2_ (PO_4_)	14	-	-	Mice (BALB/c –f)	OVA (IP)	Int T	2.7mg/kg	nc (Resist)	-	nc	nc	nc	nc	-	↑ (MIP-1); nc (IL-13, IL-4, INF-γ, TNF-α, MCP-1, Tarc, KC)	nc MUC5AC PAS	[66]
SiO_2_ (Amino)	15	-	-	Mice (BALB/c –f)	OVA (IP)	Int T	2.7mg/kg	nc (Resist)	-	nc	nc	nc	↑	-	↑ (IL-13, IL-4); ↓ (Tarc); nc (INF-γ, TNFα, MCP1, KC)	nc MUC5AC PAS	[66]
SiO_2_	100	12.7	-	Mice (BALB/c –f)	OVA (IP)	Int N	11 mg/kg x3	nc (Resist)	nc	nc	nc	nc	↑	↑	nc (IL-5, IL-13, IL-1β, INF-γ)	nc PAS	[276]
SiO_2_ (Meso)	100	70.6		Mice (BALB/c –f)	OVA (IP)	Int N	11 mg/kg x3	nc (Resist)	nc	nc	nc	nc	↑	nc	nc (IL-5, IL-13, IL-1β, INF-γ)	nc PAS	[276]
SiO_2_ (PEG)	100	12.7	-	Mice (BALB/c –f)	OVA (IP)	Int N	11 mg/kg x3	nc (Resist)	nc	nc	nc	nc	nc	nc	nc (IL-5, IL-13, IL-1β, INF-γ)	nc PAS	[276]
TiO_2_	5	-	168	Rat (BN –m)	OVA (IP)	A	9.4mg/m^3^ x6h	-	-	↓	nc	↓	↓	↓	↓ (MCP-1, IL-4, IL-6, INF-γ)	↓ PAS	[118]
TiO_2_	21	-	4200	Rat (BN –m)	OVA (In)	A	159 μg, deposited	nc (Resist)	nc	↓	nc	nc	nc	↓	-	-	[119]
TiO_2_	21	-	4200	Rat (DA-m)	OVA (In)	A	168 μg, deposited	nc (Resist)	nc	↓	↑	↑	nc	nc	-	-	[119]
TiO_2_	21	-	1.5 μm	Mice (BALB/c –f)	OVA (IP)	A	193 μg, deposited	↓ (Resist)	nc	↓	↑	↓	nc	↓	↓ (IL-4, IL-13, IL-5,IL-6, INF-γ)	-	[120]
TiO_2_	21	-	1.5 μm	Mice (BALB/c –f)	OVA (IP)	A	32 μg, deposited	↑ (Resist)	-	↑	↑	↑	↑	↑	nc (IL-4, IL-13, IL-5,IL-6, INF-γ)	-	[120]
TiO_2_	4-8	130	244	Mice (BALB/c)	OVA (IP)	Int N	5.5 mg/kg x4	nc (Resist)	-	-	-	-	-	-	-	-	[273]
MWCNT	D – 9.5, L– 1.5μm	250-300	-	Rats (BN –f)	TMA (dermal)	A	22mg/m3 x5h x7d	-	nc	nc	nc	↓	nc	nc	-	-	[68]
MWCNT	D - 20–30, L-5-10μm	-	324	Mice (C57BL/6)	HDM (Int N)	O	2 mg/kg	-	nc	nc	↑	nc	↓	↑	nc (IL-1β, IL-13, IL-4, IL-5 CCL20) ↑(CCL2, CXCL1/2)	-	[71]
MWCNT	D 30–50 L 0.3-50μm	109.29	>2 μm	Mice (C57BL/6J)	OVA (IP)	OA	4mg/kg	-	nc	↓*	nc	nc	↓	-	nc (IL-13, IL-5,, IL-17α) ↓(TGF-β1)	nc PAS	[70]
MWCNT	D 30–50 L 0.3-50μm	109.29	-	Mice (C57BL/6 –)	OVA (IP)	A	100mg/m^3^	-	-	nc	nc	nc	nc	-	nc (IL-13) ↑ (IL-5, TGF-β1)	-	[67]
MWCNT	D 10–30 L 0.3-56μm	-	-	Mice (WT –m)	OVA (IP)	OA	4mg/kg	-	-	nc	nc	nc	nc	nc	nc (IL-13, IL-1β, TGF-β1, TNF –α, IL-10)	nc PAS	[69]

Abbreviations: *Agg* agglomerate, *AHR* airway hyperresponsiveness, *LF* lung function, *Resist* Resistance, *Penh* Enhanced pause, *Eos* eosinophils, *Neu* neutrophils, *Lym* lymphocytes, MΦ macrophages, *GCH-MS* goblet cell hyperplasia-mucus secretion, −*f* –female, *OVA* ovalbumin, *IP* intraperitoneal injection, *A* aerosol, ↓ decrease, *nc* no change, ↑ increase, − not determined, −*m* –male, *Int N* intranasal instillation, *PA* pharyngeal aspiration, *PAS* Periodic acid–Schiff staining, *TDI* toluene diisocyanate, *OA* oropharyngeal aspiration, *Int T* intratracheal instillation, *PEG* Polyethylene glycol, *Meso* mesoporous, *BN* Brown Norway, *DA* Dark Agouti, *MWCNT* multi-walled carbon nanotubes, *D* diameter; *L* length, *TMA* trimellitic anhydride, *HDM* house dust mite

Early investigations into CNPs and their ability to modify pre-existing ragweed pollen induced AAI in the beagle dog, apart from a mild neutrophilic response, did not show any major effects on airway reactivity or immune activation [58]. More recent studies demonstrated a neutrophilic response when CNPs were administered in mouse models of OVA induced inflammatory disease [59, 60]. In addition, earlier studies from these same authors revealed exacerbation effects of CNPs on AHR, eosinophils, lymphocytes, IL-13 and mucus hypersecretion, which was more severe when CNPs were administered just before, rather than after allergen challenge [61, 62]. Gold NPs (AuNPs) and TiO_2_NPs have been examined using a model of occupational asthma, where exposure of toluene diisocyanate sensitised mice to either NP resulted in an increase in AAD with AuNPs showing greater effects [63]. The doses used in this study were comparably lower than the majority of other similar *in vivo* studies and aimed to reproduce occupational exposure levels.

Copper oxide NPs (CuONPs) displayed noted potency for their ability to exacerbate AHR, mucus hypersecretion and allergic inflammatory markers including IgE, IL-5 and IL-13 in murine OVA induced AAD, with dose dependent effects observed from 25 to 100μg/kg [64]. Graphene oxide (GO) is another NP examined for exacerbation effects in an allergic airway model of exposure. Repeat administration at the challenge phase of the protocol did not significantly modulate allergic endpoints but did cause significant neutrophilia [65]. Dose dependent increases in inflammatory cell infiltrates including macrophages and eosinophils were induced by SiO_2_NPs and accompanied by increased Th2 type inflammation and mucin hypersecretion [66].

Interestingly, the effects of MWCNTs in their ability to aggravate pre-existing AAD appear modest. Ryman-Rasmussen and colleagues demonstrated no effect on inflammatory cell infiltration at either 1 or 14 days post challenge exposure. There was however a modest increase in IL-5 which was suggested as a potential contributor to fibrotic events observed only with MWCNTs and OVA treatment at day 14 [67]. This lack of modifying effect was also observed in a rat model of trimellitic anhydride induced allergy [68] and a murine OVA allergy model [69] while inhibitory effects were observed for eosinophil and macrophage infiltration in another study [70]. Moderate exacerbation responses were also observed in a HDM model of AAI [71].

## Mechanistic insight into how nanomaterials may influence asthma

 An understanding of how NMs with defined characteristics influence models of asthma and AAD has the potential to identify characteristics or material types that can be linked to molecular and cellular events critical for disease initiation and exacerbation in humans. This information can be used to identify NM “properties of concern” but importantly can also be used to improve choices surrounding relevant endpoints of disease, model selection, testing approaches and prediction strategies towards a more complete assessment of NM hazard. In this part of the review we will document those studies, which have explored key mechanistic events in models of asthma and AAD suggested as underpinning NM adverse effects. This will include discussion of not just *in vivo* approaches but those attempts at modelling key events *in vitro* (Additional file 1: Table S3).

### Direct effects on sensitisation in allergic airway disease

The development of an adaptive immune response to an allergen is central to those with AAD. This process typically involves dendritic cells, which take up antigen, become activated, mature and travel to lymph nodes where they present these antigens to T and B cells to direct their differentiation to specific functional phenotypes.

The influence of NMs on this process has been examined *in vivo* and *in vitro*, with initial studies focussing on the role of the dendritic cell and T-cell interaction. Using adoptively transferred DO11.10 CD4+ T-cells in mice, which respond specifically to OVA peptides presented from antigen presenting cells, CNPs when administered to the lung in combination with OVA induced a proliferative response [72]. This T-cell response indicates increased dendritic cell (DC) antigen presentation. The number of myeloid dendritic cells (DCs) as well as the expression of DC maturation markers CD80 and CD86 in the peribronchial lymph nodes of these mice were also increased. Knockout (KO) mice deficient for these dendritic cell co-stimulatory molecules failed to produce the adjuvant effects of CNPs on AAI. A direct maturation effect of CNPs on dendritic cells *in vitro* was also demonstrated, altogether providing strong evidence that the sensitising effects of CNPs in AAD in this KO model arise from direct effects on dendritic cell maturation [72]. A size dependent accumulation of antigen presenting cells induced by CNPs in the mouse lung has also been observed, where 14nm but not 56nm sized particles increased the number of cells positive for CD80, CD86 and MHC class II [39]. Moreover, this size dependency was observed *in vitro* through the ability of 14nm but not 56nm CNPs to enhance T-cell proliferation in an allogeneic mixed lymphocyte reaction assay [73]. Direct effects of CNPs on T-cell differentiation have also been documented. Splenic leukocytes from DO11.10 transgenic mice were incubated with ovalbumin and two different sizes of CNPs (22nm and 39nm) were examined and shown to have little effect on OVA induced T-cell proliferation. However, the smaller CNPs induced the expression of Th2 cytokines including IL-4 and IL-13 to a greater extent than the larger [74]. Interestingly, cell free BAL from CNPs instilled mice can induce the maturation of bone marrow derived dendritic cells (CCR7 expression) pointing towards soluble factors released within the lung as also having a role in directing the sensitisation process [41].

In addition to CNPs other types of nanomaterials have been investigated for their ability to modify dendritic and T-cell responses. Chen and colleagues demonstrated that PEGylated SiO_2_NPs have direct effects on T-cell differentiation signals (IL-2 and IFNγ), but this was only observed for CD8+, not CD4+ T-cells [75]. The ability of SiO_2_NPs to enhance cross-presentation of dendritic cell antigens to CD8+ T-cells has also been observed [76]. Both these studies demonstrate size dependent effects and while no direct effects on allergy related CD4+ T cell differentiation were observed, it is further evidence that NPs can influence the adaptive immune response.

Studies on the mechanisms through which adjuvant activity of NPs may occur have focussed not only on how cellular behaviour and differentiation can be altered but also on what molecular interactions drive such responses. Early insight stemmed from initial observations that CNPs enhanced AAD, and that this correlated with the level of cellular oxidative damage in lung tissue [37]. This oxidative injury has also been observed with SWCNTs as increased lipid peroxidation and 8-hydroxy-2′-deoxyguanosine staining within the lung [52]. These changes were paralleled by an increase in the maturation of bone marrow derived dendritic cells and antigen-specific syngeneic T-cell-stimulating capacity *in vitro*. However, it was not determined which cell type accounted for oxidative injury or what characteristic of the SWCNTs may be responsible for these changes [52]. A role for oxidative injury in SWCNTs mediated worsening of AAD development and adjuvant activity was also suggested from the observations that vitamin E co-administration attenuated oxidation biomarkers and the detrimental effects of the NP [53]. Again however, there was no suggestion as to the precise cellular and molecular events underlying these observations.

Oxidative surface chemistry and increases in reactive oxidant species (ROS) within living systems are not necessarily directly related but have been put forward to explain differential dendritic cell maturation and T-cell responses. Human primary monocyte derived dendritic cells were exposed to either TiO_2_NPs or cerium dioxide NPs (CeO_2_NPs) identified as having oxidative and reductive surface chemistry respectively. TiO_2_NPs induced cellular injury was accompanied by DC maturation and increased expression of CD83, CD80, CD86 and CCR7 together with the induction of IL-12 and TNF-α. CeO_2_NPs on the other hand did not result in any significant changes in cellular viability or maturation markers, but did produce IL-10 and IL-6. T-cell responses to these NP treated dendritic cells (DCs) were also examined, with TiO_2_NPs inducing a profile of Th1 cytokine production (IL-2, IFN-y) and CeO_2_NPs inducing a Th2 response (IL-4, IL-5, IL-10). The authors suggest that the oxidative capacity of TiO_2_NPs was responsible for increased ROS mediated inflammasome activation (IL-1β), DC maturation and differentiation of T-cells towards a Th1 profile. They also suggest, in contrast that the CeO_2_NPs, which induced anti-inflammatory IL-10 and Th2 biased responses, could be attributed to the anti-oxidant surface chemistry of this NP [77]. While this is an interesting set of observations, it cannot be ruled out that surface chemistry independent effects played a role.

The effects of PSNPs on AAD have also been attributed to alterations in DC function. These NPs (PS50G; glycine coated 50nm size) when administered 12 days before sensitisation to OVA resulted in an inhibition of AAI, mucus hypersecretion, allergen specific IgE levels and Th2 cytokines in the lung draining lymph nodes [78], effects not observed with larger sub-micron sized particles of the same material [79]. This inhibition was not attributed to effects on sensitisation but rather DC function at the challenge phase, explained by a reduction in the numbers of allergen-containing CD11b^hi^MHCII^hi^ DCs in the lung and CD11c^+^MHCII^hi^ DCs in the lung and draining lymph nodes. An additional mechanism was suggested as PS50G abolished the ability of CD11b^+^ DCs to induce OVA specific CD4+ T-cell proliferation [78]. Further investigation into these effects demonstrated that PS50G induced DC recruitment and maturation through cytokine induction in the lung but with a reduced number of stimulatory DCs translocating to the lymph node [79]. Interestingly the authors have suggested that given the timescale of NP administration 12 days before the start of sensitisation, DC refractoriness as a consequence of activation in the absence of OVA may account for the inability to mount an adaptive immune response at the challenge phase [78]. Furthermore, it was speculated that evolutionary conserved responses to viral sized particles, which would aim to limit inflammatory reactions not focussed on anti-viral immunity (e.g. Th1 T-cell activation) such as Th2 type adaptive immune responses, may be activated by the nanoscale properties of NPs and contribute to the inhibitory effects. This can be supported by observations that PS50G did not result in inhibition of allergen specific lymph node production of IFNγ or have any effect on Th1 anti-viral immunity in an acute lung influenza virus infection model [78].

The inhibitory effects of PS50G on AAI also did not extend to inhibition of AHR [78]. This disparity between NP effects on allergic inflammation and AHR has been observed for other types of NPs. Graphene oxide (GO) augments AHR, goblet cell hyperplasia and smooth muscle hypertrophy in OVA induced allergic airway disease while at the same time inhibiting Th2 cytokine production, eosinophilia and IgE levels [65]. The mechanism put forward to explain this suggested that increased macrophages within the lung and their production of chitinases such as CHI3L1 contributes to airway remodelling/hyperresponsiveness and favours the development of Th1 over Th2 type responses. These observations were supported by increased levels of OVA specific IgG2a [65]. Inhibition of adjuvant activity and AAI has been observed for other nanomaterials, including Fe_2_O_3_ [80] and SiO_2_ coated TiO_2_ [81] where they were found to have size and dose dependent effects. While the authors speculate that the mechanism through which this inhibition occurred involved dendritic cell modification, modulation of the balance of Th1/Th2 T-cell responses and direct effects on regulatory T-cells, no supporting evidence was offered.

Aluminium salts, the most commonly used adjuvant in humans, have been well characterised for their ability to promote the development of adaptive immunity to co-administered allergens, such as OVA, producing a predominant Th2 type immune response [82]. The classic understanding of how these adjuvants have their effects has been described in terms of their ability to aggregate antigens, stabilise protein structure, prevent degradation and to provide a pool of material for continuous release to antigen presenting cells [83]. However, recent work has suggested that other mechanisms are involved as a likely consequence of direct cellular injury, including the activation of innate immune responses and the recruitment of inflammatory cells. Activation of the NLRP3 inflammasome as a consequence of particulate lysosomal overload in resident phagocytes (including macrophages and dendritic cells), uric acid release and subsequent caspase-1 activation and release of inflammatory cytokines, such as IL-1β, IL-18 and IL-33, have also been suggested as important events underlying adjuvant activity [84, 85].

Given the particulate nature of NPs it is perhaps unsurprising that these same mechanisms have been suggested to underlie NP modifying effects on adjuvancy [86]. Particular attention has been given to the potential role of the NLRP3 inflammasome, with nanomaterials including AgNPs, TiO_2_NPs, SiO_2_NPs and CNTs all inducing markers of this pathway’s activation [87–89]. Using gene KO approaches, a direct role for the NLRP3 inflammasome in mediating the IL-1β response to TiO_2_NPs in macrophages and dendritic cells has been demonstrated. IL-1α but not NLRP3 however, was identified as a major control point for neutrophilic responses after instillation in murine lung [90]. Nano sized particulate forms of TiO_2_ and SiO_2_ were observed to have a greater impact than submicron sized particles of the same material on the maturation of bone marrow derived dendritic cells. Using KO mice, IL-1β responses to these NPs in dendritic cells were found to be NLRP3 and caspase-1 dependent [91]. Mechanistic insight into how NPs may preferentially activate the inflammasome to induce release of IL-1β and IL-18 in dendritic cells stemmed from observations that production of these mediators was greater with 30nm SiO_2_NPs than larger nano or sub-micron sized particles. The authors went on to demonstrate size dependent effects on cellular uptake, ATP release and ROS production and through interventional studies suggest a sequential mechanism involving NP uptake, lysosomal injury, ATP mediated activation of purinergic receptors and NADPH oxidase dependent production of ROS as a terminal signalling event for inflammasome activation [92].

Other mechanisms of NP effects on the adaptive immune system include examples where exosomes were induced in the alveolar region after inhalation of magnetic iron NPs. These exosomes incorporate antigen for systemic delivery to antigen presenting cells such as dendritic cells and modulate T-cell differentiation towards a Th1 T-cell phenotype [93]. While Th2 and Th17 immune responses are more commonly associated with asthma it is still of considerable interest that modulation of the immune system in this way by NPs can occur. Furthermore, it is interesting to speculate as to whether other types of material can induce exosome formation to direct other types of hypersensitivity reactions.

Th17 inflammation and the neutrophilic response are a significant component within the asthma phenotype particularly in those with severe asthma [94, 95]. Through the use of genetic KO and cell depletion models, evidence points towards a direct role for neutrophils in controlling AHR [96]. Whether NP induced neutrophilic responses contribute to AHR and modify Th17 responses is relatively unexplored. One study using the chemical compound ectoine, which preferentially reduced BAL neutrophils (with no changes in other BAL cells), suggested that the protective effects of this compound on CNPs adjuvancy (IgE, Th2 profile) may be attributed to the reduction in neutrophils [41].

Lastly, the airway epithelium has been observed to play a central role in the development of sensitisation in asthma, mainly through the ability to detect allergens and associated injury and respond by producing signals such as TSLP, GMCSF, IL-25 and IL-33, which target dendritic cells to initiate sensitisation mechanisms [97]. Examples of how NPs can impact on this process include an *in vivo* HDM induced allergy model where the airway epithelium was proposed as the primary source for signals governing airway sensitisation in response to MWCNTs [51]. Similar suggestions have been made for IL-33 production in the murine lung by MWCNTs in an OVA induced AAD model [49]. 16HBE14o- airway epithelial cell exposure to CNPs and TiO_2_ NPs resulted in an increase in GMCSF, which was suggested as a consequence of either NP intrinsic capability or cellular induction of oxidative stress depending on the material type, and that this effect was size, surface area and cellular internalisation dependent [98]. SiO_2_NPs have also been observed to induce GMCSF from BEAS2B airway epithelial cells [99].

### Insight into the impact of nanomaterials on established allergic airway disease

With the shift to Th2 type adaptive immunity in allergy, B-cell class switching to IgE production and its subsequent release occurs. This is followed by FCΕRIα receptor binding in cells and tissues resulting in inflammatory responses when allergen is reencountered. In atopic individuals, allergen binding to IgE present on mast cells and basophils induces the release of pre-existing pro-inflammatory mediators such as histamine, proteases, proteoglycans and cytokines [100, 101]. This process is a central mechanism for how allergen induced asthma exacerbations manifest at a cellular level.

A number of studies have examined how NPs can impact mast cell function. TiO_2_NPs have been demonstrated to induce the degranulation of RBL-2H3 mast cells with the release of histamine, in an oxidative stress and calcium flux signalling dependent manner [102]. This effect was observed without any IgE mast cell priming and suggests that these NPs may directly activate mast cells *in vivo* independent of allergen. Similar findings were observed with AgNPs where degranulation of murine bone marrow derived mast cells was also examined for effects independent of IgE. The smaller sized 20nm but not 110nm sized particles resulted in degranulation, which was found to be dependent on scavenger receptor signalling, indicating cellular uptake as important. Extracellular Ag^+^ ions did not have any effect [103]. Interestingly, pulmonary instillation of CeO_2_NPs induced inflammatory cell and mediator production, which was not observed in mice deficient in mast cells. This study also examined bone marrow derived mast cells and found that CeO_2_NPs induced inflammatory mediator production from IgE/allergen primed cells but did not induce degranulation [104]. As observations of the effects of nano-sized materials on asthma exacerbation also include inhibitory or protective effects, it is interesting to note examples where attempts have been made to interrogate how this may occur. Fullerene derivatives have been observed to stabilise mast cells, prevent IgE dependent activation and to attenuate disease parameters in both exacerbation and sensitisation models of asthma [105, 106]. These fullerene derivatives possess inherent anti-oxidant capabilities derived from their ability to catalytically scavenge large numbers of oxygen free radicals [107] and this has been suggested as a mechanism through which they elicit their biological effects. This material has also been reported to increase production of P-450 derived cis-epoxyeicosatrienoic acids, eicosanoid metabolites in the lung, which stabilise and prevent IgE mediated mast cell degranulation [106]. SWCNTs were also observed to inhibit IgE mediated RBL2H3 degranulation. In this study the authors pointed to structural similarities to fullerene derivatives but in the absence of a demonstrated ROS scavenging capability of SWCNTs the mechanism underlying this attenuating effect is yet to be determined [108]. ZnONPs were found to inhibit OVA induced degranulation of OVA specific IgE primed RBL2H3 cells, which was not observed with larger sized ZnO particles. These effects were correlated with levels of intracellular Zn^2+^ ions, and administration of ZnSO_4_ also produced an inhibitory effect on mast cell degranulation. ZnONPs have also been observed to inhibit both basal and IgE mediated degranulation of RBL2H3 and bone marrow derived mast cells, which was observed to a much greater extent for the 30nm rather than the 200nm sized particles. In contrast, TiO_2_NPs examined in the same study, produced a modest increase in mast cell degranulation. Observations that smaller particles produced a greater increase in intracellular Zn^2+^ ions and a decrease in Ca^2+^ concentrations than larger particles was suggested as a mechanism through which degranulation was inhibited [109].

Similar to investigations of sensitisation and AAD development, oxidative and electrophilic stress has also been put forward as a molecular control point for exacerbation of pre-existing AAD. Inhalation of CNPs, for example just prior to allergen challenge *in vivo* resulted in exacerbation of allergic inflammation and AHR, which was attenuated by systemic administration of the anti-oxidant compound N-acetyl cysteine [110]. CuONPs have also been reported to increase disease associated ROS levels in a murine model of OVA induced AAI, which correlated with the degree of exacerbation [64]. NPs can have pro-oxidant functional groups, can possess redox cycling ability and may generate ROS from perturbation of cellular processes such as the mitochondria. Determining whether a NP has any or all of these characteristics will be important for attempts at classification of materials likely to contribute to asthma exacerbations in humans.

In addition to fullerene derivatives [105, 106], anti-oxidant capabilities have also been suggested as a mechanism of inhibition of OVA induced AAD by AgNPs [111]. The authors in this study did not identify cellular targets for the attenuating effects on ROS levels and disease parameters, or whether inherent anti-oxidant properties of AgNPs or the prevention of cellular events leading to ROS generation were responsible for the observed inhibitory effects. Using the same model in a follow on study the authors suggest signalling molecules including HIF-1α and RTK signalling as possible contributors [112]. In contrast, AgNPs have been observed to induce ROS in cell free [113] as well as upon interaction with cellular [114, 115] systems, and have been demonstrated to induce oxidative stress in other investigations of OVA induced murine AAD [116]. The reasons for these differential effects with AgNPs could be attributed to particle differences such as size, as well as model specific parameters including dose effects. The inhibitory effects of AuNPs on AAD have been reported in a study by Barreto and colleagues, using two different mouse strains, an effect that was associated with a decrease in ROS generation in BAL cells [117]. One similarity between these studies on AuNPs and AgNPs was that the smaller primary-sized particles (6nm) of both materials were associated with inhibitory effects, while the larger-sized was associated with exacerbation effects.

TiO_2_NPs have been observed to reduce AAI in rats with pre-existing disease. Here it was argued that a direct effect on Th2 type inflammation was the mechanism through which this effect manifested. It was also suggested that the lack of inhibitory effects in other studies, could be attributable to different routes of administration (aerosol versus instillation) causing different agglomeration, deposition patterns and biological responses within the lung [118]. Inhibitory effects of TiO_2_NPs on AAI were also observed in different rat strains [119]. This was paralleled by TiO_2_NPs induced increases in neutrophil and lymphocyte pulmonary responses in dark agouti rats, which are predisposed to chronic inflammatory disorders, but not in Brown Norway rats, which are pre-disposed to allergic responses. [119]. In addition to these effects, another study demonstrated that a single dose of TiO_2_NPs given at the challenge phase resulted in an exacerbation of AAI and AHR. Repeat dose exposure in the same model, while resulting in reduced allergic inflammatory markers and AHR, resulted in a marked neutrophilic response and body weight loss pointing towards effects associated with general health decline rather than any protective response attributable to the particles [120]. The authors also suggest that activation of the inflammasome via ROS generation may contribute to the responses observed in this study.

Surface chemistry has been proposed as an important determinant of NM effects on biological systems. Uncoated SiO_2_NPs were observed to exacerbate AAD in an OVA-induced mouse model. When the NPs were coated with either amino or phosphate groups this exacerbation effect was attenuated. Macrophage activation markers were not induced by the modified particles and it was suggested that recognition and uptake of the NPs by macrophages were attenuated potentially as a result of protein corona differences in the NPs. Other mechanisms suggested for the lack of effect with coated materials include a modified ability to affect the thermodynamic characteristics of lipid monolayers, including lung surfactant and cell membranes [66].

Additional mechanisms for how NM may affect those with pre-existing AAD include the ability to enhance the presentation of allergens. The HDM allergen DERP1 when coated onto AuNPs produced stronger basophil activation from allergic patients compared to exposure to the same amount of free allergen and was correlated with protease activity of the allergen [121]. In addition to activation and degranulation of mast cells and basophils as part of exacerbation events, direct effects of NPs on neutrophil and eosinophil responses have also been investigated. Primary human peripheral blood derived neutrophil degranulation was significantly induced by TiO_2_NPs and CeO_2_NPs with ZnONPs producing a more modest effect. Interestingly these effects were more potent than the classical bacterial FMLP agonist [122]. A number of other studies have demonstrated activation of neutrophils by NPs, with intracellular events such as calcium signalling proposed as key in the induction of a respiratory burst and inflammatory mediator transcription and release. One such study investigated the sub-lethal impact of different NPs on HL-60 neutrophil like cells and found that AgNPs but not TiO_2_NPs, ZnONPs, CNPs or MWCNTs induced increases in intracellular Ca^2+^ levels [123]. Direct activation of eosinophils was observed for ZnONPs and 20nm AgNPs but not CeO_2_NPs, TiO_2_NPs and 70nm AgNPs [124]. ZnONPs were also observed to have similar effects in primary human eosinophils in another study from the same group [125]. Whether such effects are relevant for modulation of exacerbation in AAD has yet to be determined.

Regulation, recruitment and control of immune and inflammatory signalling is becoming more of a focus for how NMs elicit biological responses and contribute to exacerbation of airway disease. Interventional strategies including gene KO approaches have been used to interrogate how particular inflammatory signalling events and pathways contribute to NM exacerbation effects. Using such knockout approaches, MWCNTs were shown to exacerbate OVA induced AAI through a mechanism involving cyclooxygenase 2 (PTGS2) activity, an enzyme that controls levels of prostaglandins, key regulators of immune system responses [70]. In addition, the ability of MWCNTs to cause exacerbation of AAI *in vivo* was further enhanced through KO of the STAT1 gene [69], a transcription factor important for mediating interferon and Th1 signals, immune cell signals suggested as having protective effects in allergic asthma. Similar to effects on sensitisation, exosome formation in the alveolar region after inhalation of magnetic iron oxide (Fe_2_O_3_) NPs, has also been suggested as a mechanism through which regulation of Th1 T-cell responses are guided in exacerbation of AAI [93]. Lastly, MWCNTs administered to mice were found to produce a strong neutrophilic response, which was diminished in animals with pre-existing HDM induced AAI [71]. MWCNTs did not alter the level of eosinophils or other Th2 inflammatory markers but did result in a more severe airway fibrosis. Decreased IL-1β levels together with *in vitro* investigations in this study suggest that an allergic inflammatory environment inhibits inflammasome activation through a STAT6 mechanism and that this may be responsible for the exacerbated fibrotic response observed with MWCNTs exposure [71].

### Mechanisms of asthma development and exacerbation independent of allergic sensitisation

Modelling of asthma using rodent allergic airway disease models as the prototypical testing paradigm does not reflect all types of asthmatic disease in humans. Mechanistic insight into how environmental exposures may influence disease is therefore, potentially overshadowed by predominant discussion of how materials affect allergic responses and Th2 type inflammation. A summary of those studies with observations independent of allergy driven disease identified within our search parameters are documented in Additional file 1: Tables S1 and S2. Those studies primarily focussed on allergen independent models are summarized in Additional file 1: Table S1, while those observations independent of allergen effects but documented as part of larger allergen driven model studies are summarized in Additional file 1: Table S2.

Some of the earliest studies to investigate the pulmonary effects of nanomaterial exposure did not specifically set out to model the potential impact on disease conditions such as asthma. However observations including neutrophil and inflammatory mediator alterations can be viewed not just in terms of broad pulmonary toxicity effects but as having the potential to influence disease initiation and progression in conditions such as asthma and AAD. One of these early studies set out to compare similar sized metallic NPs after pulmonary instillation in rats. It was demonstrated that the level of toxicity and neutrophil response was material specific with nickel (Ni) and cobalt (Co) having the greatest effects. It was suggested that the surface chemistry of these materials as expressed in terms of their ability to generate reactive oxygen species, governed specific potency [126, 127]. Other factors including NP solubility and surface area have been put forward as the most significant characteristics influencing NP toxicity after pulmonary exposure and have been reviewed extensively elsewhere [128, 129].

In a clinical setting, treatment strategies targeting Th2 type inflammation reduce allergic exacerbation rates but have little effect on baseline measures of asthma activity such as AHR [130]. These and other observations support the suggestion that asthma should be considered as a condition with a fundamental abnormality in smooth muscle function that underlies diminished lung function and AHR [130, 131]. The concept that AHR and allergic inflammation are not exclusively linked is consistent with observations in this review and has been discussed previously. Indeed, direct effects on AHR have been observed in the absence of allergic airway modelling for AgNPs, TiO_2_NPs, SWCNTs and MWCNTs exposures [132–136]. While an understanding of how such effects on AHR manifest is unknown at this time, it is clear that smooth muscle cells are likely to have a central role [137]. The direct impact of nanomaterials on smooth muscle has been explored to a limited extent. TiO_2_NPs instillation in mice resulted in changes in lung gene expression consistent with effects on ion homeostasis and smooth muscle contractibility [138]. *Ex vivo* examination of AgNP applied directly to rat tracheal rings found that the nanoparticles caused a non-reversible contractile response to acetylcholine [139]. It was suggested that these NPs may interact with and modify muscarinic receptors on smooth muscle and induce nitric oxide as a mechanism to directly influence contractility. Additional observations using *ex vivo* smooth muscle preparations have also revealed increased contractility on exposure to SnO_2_NPs and CoFe_2_O_4_NPs [140, 141]. *In vitro* modelling of smooth muscle cells and exposure to NPs has also been attempted to a limited extent. AuNPs were observed to directly alter plasma membrane potential and intracellular calcium signalling in human airway smooth muscle cells in a charge dependent manner [142]. Intracellular calcium signalling is intimately involved in muscle contraction and suggests such direct modifications by AuNPs could impact contractility and ultimately AHR. An examination of different NP effects on human airway smooth muscle cell mechanical function after direct exposure, using optical magnetic twisting cytometry revealed material specific, dose and size dependent effects [143]. These included CuONPs, which inhibited smooth muscle cell stiffness, histamine contractility and isoproterenol relaxation, while larger micron sized CuO particles did not.

In addition to smooth muscle cells, airway remodelling and AHR in asthma are associated with peribronchial fibrosis and fibroblast expansion [144]. Nanomaterial influence on this aspect of airway disease has revealed direct effects, including the ability of graphene oxide, NiNPs and MWCNTs to increase extracellular matrix deposition after pulmonary instillation [145–148]. This profibrotic effect within the lung has also been observed for CeO_2_NPs, ZnONPs and SWCNTs [148–151]. The involvement of the innate immune system may also play a role here. An investigation into MWCNTs effects suggests that airway epithelial signals initiated by inflammasome activation induce fibroblast proliferation and pro-fibrotic gene expression [152]. IL-33 is an epithelial cell derived innate immune signal, which can influence type 2 inflammatory events. Knockout (KO) of IL-33 in mice causes an inhibition of the inflammation, peribronchial fibrosis and AHR induced by MWCNTs [153]. The STAT1 transcription factor, which is involved in intracellular inflammatory signalling, also contributes to MWCNTs induced airway pro-fibrotic exacerbation in mice [69]. Further examination of NP effects in pre-existing allergic airway disease revealed that SWCNTs and MWCNTs also induce airway fibrosis [53, 67]. Whether allergic inflammation was necessary for these later effects was not fully investigated. Airway epithelial cells have been argued as central players not only in directing inflammation but also in the control of fibrotic signalling in response to inhaled material. In addition to fibroblast to myofibroblast transition, epithelial to mesenchymal transition (EMT) in response to MWCNTs exposure has been proposed to contribute to pulmonary fibrosis. [154]. Instillation of MWCNTs in the mouse lung also resulted in peribronchial as well as alveolar fibrosis in another study and a role for epithelial mesenchymal transition was also suggested [155]. While a significant body of work has focussed on MWCNTs, there is a distinct lack of interrogation of other types of particles for their effects on airway remodelling and fibrosis.

Airway hyper-responsiveness and excessive mucin production are the principal mechanisms contributing to airway obstruction in asthma. The airway epithelium is the primary site for mucin release and is therefore an essential focus for the examination of nanomaterial effects. A recent study investigating the effects of NPs on mucin rheology found that positively charged polystyrene nanoparticles impaired mucin gel swelling causing mucin aggregation [156]. It was suggested that NPs with these properties would impinge mucociliary clearance and contribute to adverse disease outcomes in conditions such as asthma. Studies to date have shown induction of mucus secretion accompanied by AHR independent of allergic sensitisation for CuONPs [64] and MWCNTs [134]. It is unclear how nanomaterials cause these effects. Whether it is a result of direct action on epithelial cells, secondary signalling events from inflammatory and other resident cells or a combination of all is still unknown. Some insight however has emerged from observations that cytokines including IL-13, Il-17A and IL-1β can induce mucin production from airway epithelial cells. For example, in a rat pulmonary exposure study, it could be argued that TiO_2_NPs induced goblet cell hyperplasia and MUC5AC expression may be attributable to IL-13 produced from mast cells [157]. Direct effects on airway epithelial cells through ROS and calcium signalling was also suggested to control TiO_2_NPs induced mucin secretion [158]. Additional signalling events that may be important in the control of mucin production include airway epithelial MAPK signalling, an observation documented for CuONPs induced MUC5AC in H292 epithelial cells [159].

Airway epithelial cells are a primary target for inhaled material deposition and a control point for the recruitment of inflammatory cells. Chemokines such as IL-8, MCP1 and CCL28 act to recruit cells including neutrophils, monocytes, lymphocytes, eosinophils and dendritic cells. Production of these mediators from airway epithelial cells has been demonstrated in response to NPs including CNPs, TiO_2_NPs, MWCNTs, CeO_2_NPs, SiO_2_NPs, CoNPs and ZnONPs [160–167]. A greater understanding of these primary events in nanomaterial exposure and airway response is likely to lead to a more complete mechanistic understanding of the potential to contribute to asthma and airway disease.

Mast cells are an important part not only for atopic but also non-atopic asthma [168]. Exposure of mice to MWCNTs resulted in inflammatory responses and pulmonary functional changes similar to those observed in asthma, which were dependent on the presence of mast cells and their ability to respond to IL-33 [169]. Additional investigations demonstrated a role for the IL-33/ST2 axis together with IL-13 signalling in MWCNTs induced AHR and inflammatory events, which were independent of T and B cell involvement [135]. The authors suggest type 2 innate lymphoid cells (ILC2) recruited to the airways as a consequence of epithelial injury and IL-33 release as the primary events governing adverse effects. As these cells have the ability to produce type 2 cytokines such as IL-4, IL-5 and IL-13 in the absence of any adaptive immune response, they also represent an important target for how nanomaterials may impact asthma and obstructive airway disease [170]. Other innate lymphoid cells such as ILC3 cells, which produce IL-22 and IL-17, through inflammasome activation, may also be important as they have been directly implicated in the mechanisms of AHR induction in the absence of adaptive immunity [171].

It is recognised that respiratory tract viral infection, mainly from rhinovirus (RV), is associated with the majority of asthma exacerbations in children and more than half of adults [172]. The presence of Th2 type inflammation and asthma has also been associated with reduced antiviral defence in plasmacytoid dendritic cells and the airway epithelium, indicating increased susceptibility to viral mediated exacerbations in those with allergic inflammation [173, 174]. Despite this being a primary mechanism for asthma exacerbation, only a handful of investigations have been directed towards understanding how nanomaterial exposure, including ultrafine components of air pollution, may impact respiratory viral infections relevant for asthma. This is also true for the development of asthma. One of the earliest studies that did investigate such interactions focussed on mice exposed to CNPs prior to respiratory syncytial virus (RSV) infection [175]. This virus causes lower respiratory tract infection and bronchiolitis and the severity of infection has been associated with the development of asthma. Despite alterations in the inflammatory response, including induction of Th2 markers such as IL-13, there were no significant effects of CNPs on RSV replication or viral clearance [175]. In a similar study from the same group, CNPs were administered to mice after RSV infection and despite there being no difference in viral titre and clearance, there was an increase in pulmonary inflammation with CNPs, which was correlated to an increase in AHR [176]. Similar to CNPs, TiO_2_NPs when administered prior to RSV infection in mice did not alter viral titres but did exacerbate inflammation and pneumonia [177]. Gold nanorods interestingly have been observed to inhibit RSV infection levels in mice and airway epithelial cells, which correlated with the induction of antiviral gene expression and modulation of pattern recognition receptors [178]. Other nanomaterials including SWCNTs, AgNPs and polystyrene NPs have all demonstrated modifying effects on viral infectivity or inflammatory consequences of respiratory viral infection [179–181]. These later studies however did not focus on viruses typically associated with asthma.

### Insight from nanomedicine approaches to vaccines and allergy

NPs intended for medical use have also been investigated for modulation of sensitisation. PVA coated super-paramagnetic iron oxide nanoparticles (PVA-SPIONs) caused an inhibition of monocyte derived dendritic cells’ ability to process antigen and stimulate CD4+ T cells, without affecting antigen uptake or markers of maturation. It was suggested that PVA-SPIONs induced reversion to a more immature dendritic cell phenotype involving the regulation of lysosomal function as a potential mechanism of action. PVA-SPIONs have a slightly positive charge and therefore bind proteins with an isoelectric point (pI) of > 5.5. While not tested, it was suggested that NPs may bind proteins with a higher pI involved in antigen processing, such as Cathepsin B, H, L1 and DMB, and subsequently sequester their function, as a mechanism for how they attenuate dendritic cell function [182]. The use of NMs in the development of novel vaccines has contributed significantly to our understanding of how NMs direct dendritic cell function and sensitisation to antigen. Supporting a role for particle charge in this process, a number of studies have observed that cationic NPs produce superior adjuvant responses following pulmonary delivery, when compared to anionic particles of the same material. Pulmonary immunization using OVA conjugated cationic rod shaped hydrogel NPs led to increased antibody titres, B-cell expansion and increased activation of CD4+ T-cell populations in the draining lymph nodes. These effects were not observed using anionic particles of the same material. *Ex vivo* treatment of dendritic cells with these cationic conjugated particles induced dendritic cell maturation markers and robust antigen specific T-cell proliferation, effects not observed with anionic particles or with OVA alone [183]. In a follow on study from the same group, it was demonstrated that these positively charged NPs are preferentially taken up by dendritic cells (DCs) in contrast to negatively charged NPs, and were associated with an increased expression of the chemokines Ccl2 and Cxcl10, which possibly contribute to recruitment of Th2 promoting CD11b DCs to the lung [184]. Using the same approach as the previous hydrogel studies to modify NP surface by the addition of chemical species for negative carboxyl (−COOH) and positive amino (−NH2) charge, a study examining the effect of PVA coated AuNPs found that after instillation into the lung, cationic particles were preferentially taken up by all antigen presenting cells including macrophages and DCs. This was accompanied by an increase in CD4+ T-cell OVA proliferative responses in lung draining lymph nodes, an effect not observed with the anionic NPs [185].

Interestingly, rapid translocation of NPs to the lymph nodes after pulmonary instillation, suggested as independent of cell mediated transport, in addition to being influenced by charge is highly size dependent [186]. Size dependent preferential uptake of NPs by dendritic cells and translocation to lymph nodes was accompanied by enhanced T-cell responses when compared to larger sized particles [187]. These studies have suggested an optimal NP uptake size of between 20 and 50nm for lymph node homing migratory DCs and other antigen presenting cells after pulmonary administration. In contrast, other studies have suggested roles for larger particles in adaptive immunity modulation. As part of strategic approaches to improve vaccine development, one study examined how antigen conjugated to particles in the viral size range influenced induction of adaptive immune responses. Intranasal administration of larger 200nm pluronic-stabilized poly(propylene sulphide) NPs (PSPNPs) were observed to deliver OVA antigen more efficiently into both MHC I and II presentation pathways and increase the number of poly functional CD+ T-cells when compared to the smaller 30nm size [188]. Moreover, immunisation approaches using different size OVA conjugated PS beads administered intradermally found that larger sized beads (93nm, 101nm & 123nm) induced IL-4 release from spleen derived CD4+ T-cells *ex vivo*, while smaller sized (40nm, 49nm) induced the release of IFN-γ from CD8+ T-cells. This size dependent effect on adaptive immunity patterning was suggested to be a direct result of modification of dendritic cells [189].

Immunotherapy is an established treatment for allergies. New approaches using viral like particles (VLPs) loaded with TLR ligands such as unmethylated CpG, targeting antiviral innate immune responses have the potential for more effective treatment in the absence of side effects associated with other strategies [190]. It is proposed that the advantages of this approach stem from the nanosized structure of VLPs, allowing transport to lymph nodes where they can be taken up by resident DCs. Processing then allows endosomal TLR9 activation by unmethylated CpG and the subsequent patterning of T-cells towards a Th1 phenotype, which in turn competes and/or inhibits Th2 type allergic responses [190]. It has been suggested that improvements to the nanocarrier approach to delivering unmethylated CpG within the lung may further enhance the ability of this TLR ligand to modulate and inhibit allergy and to overcome aspects of efficacy of VLPs observed in preclinical and clinical trials. One approach involved conjugation of unmethylated CpG to the surface of PSPNPs, where intranasal administration prior to sensitisation significantly reduced AAI and IgE levels in a murine model of HDM induced disease [191]. It was suggested that surface associated, rather than encapsulated CpG (observed with VLP approaches) may allow a more robust approach to modifying adaptive immunity towards Th1 responses. This mechanism of delivery for these nanomaterial associated pathogen associated molecular patterns (PAMPs), towards antigen processing, presentation and adaptive immune response influence, is one which should be considered carefully as a significant component to allergy development and exacerbation events. Indeed, the bacterial cell component and TLR4 agonist lipopolysaccharide (LPS) is necessary for the induction of metal allergy to Ag when administered as AgNPs [192]. Ag^+^ ions and metal NPs of low solubility failed to induce allergy under similar conditions. These observations again highlight the importance of nanostructure and co-exposure to pattern recognition receptor (PRR) ligands in allergy development. Interestingly, in addition to being suggested as a central mechanism for how adjuvants can enhance and direct adaptive immune responses [83, 85], PRR activation has been observed directly by NPs. TLR4 binding and activation by TiO_2_NPs for example has been observed in pulmonary epithelial cells [193] and using a TLR4 KO murine inhalation model system was also demonstrated to mediate ZnONPs pulmonary inflammatory effects [194].

## Ultrafine pollutant nanomaterial exposure and asthma

Air pollution is one of the greatest non communicable disease related public health concerns. An estimated 3.3 million premature deaths worldwide per year have been attributed to outdoor air pollution with the particulate fraction playing a major role [195]. Respiratory health effects are among the primary concerns, with asthma accounting for a large proportion [196]. Evidence for an association between air pollution and in particular traffic derived material, and asthma has accumulated over the last decades, with systematic reviews continuing to build a weight of evidence for direct causal effects [20, 196–199]. The precise nature of the exposure material(s) and the mechanisms underlying these associations is not complete and further understanding may allow more targeted and efficient strategies to reduce adverse health effects. The ultrafine particulate (UFP) component of pollutant exposure has been highlighted as a particular focus for investigation due to unique physicochemical properties and interactions with biological systems. It is the aim of the discussion in this section, to document evidence for the role ultrafine particulates (UFPs) may play in asthmatic disease and the potential mechanisms involved.

### Human ultrafine and nanomaterial exposure effects

Most of the information on human exposure to nanoscale materials and their influence on asthma has originated from the study of pollutant ultrafine material. The majority of particles in terms of numbers within urban pollutant particulate matter (PM) are found within the UFP fraction (typically described as <100nm diameter), while most of the mass is found in particles >100nm in size [200]. One of the earliest epidemiological studies in asthmatic adults found that associations between the UFP fraction and asthma outcome, assessed using lung function parameters (Peak Expiratory Flow (PEF)), were greater than those of the fraction between 0.1-10μm (PM10) [200]. A follow on study from the same research group in an independent adult asthmatic population, found associations between cumulative (up to 14 days) exposure to both fine (<2.5μm (PM2.5)) and UFP fractions and asthma steroid medication use. It was however, difficult to separate out the effect of UFP material on asthma symptoms from other pollutant components, including NO_2_ and larger particulates [201]. An association between UFP levels and asthma incidence has also been observed in an urban environment but again confounding factors including other pollutants cannot be ruled out [202]. A separate series of studies examining air pollutant exposure in a Finnish adult asthmatic population found an association between a larger particle number and decreased lung function (PEF), which they suggested could be attributed to the level of UFPs. Yet, they did stress that the associations could not be entirely separated from the potential effects of other pollutants such as SO_2_, NO_2_ and CO [203, 204]. Separation of the UFP effects of air pollutant material on asthma has been observed to some extent, however, in a study examining lung function (forced expiratory volume-one second (FEV_1_) and forced volume capacity (FVC)) in adult asthma sufferers after a 2hr walk in either high or low air pollutant environments. In this study significant associations between higher levels of atmospheric pollutants and reduced lung function were observed for UFP and carbon content, which were not observed to the same extent for PM2.5 or NO_2_ levels. These effects were more pronounced in those volunteers with moderate versus mild asthma [205]. Severe asthma sufferers were not recruited in this study. A similar 2hr walking adult volunteer study found associations between lung function decline and higher levels of pollutant material, the effects being greater in those with severe asthma. In this study however it was not possible to separate out the effect of UFPs from other pollutants [206].

Studies in paediatric asthmatic populations have also been carried out. One early examination failed to distinguish any association of UFPs with diminished lung function as distinct from other pollutant material [207]. Indeed, more recent observations have found that nitrogen oxide pollutants, coarse and fine particles but not UFPs in urban air correlated with hospital admissions for asthma in children [208]. There are however other studies that do suggest associations between UFPs and reduced lung function/respiratory problems/asthma in children. Paediatric visits to health care facilities for acute asthmatic events requiring prednisolone were significantly associated with UFP levels in the previous 7 days. This association was also found for CO levels but not for larger particles, carbon black, ozone or SO_2_ [209]. Similarly, a separate study demonstrated paediatric asthma hospital admissions were associated with ambient urban background levels of total particle numbers, UFPs (mass) and NO_2_ but not PM10 (mass) [210]. When examining wheezing in infants, researchers found a positive correlation with increased UFPs in those below 1 year. Caution was expressed, however, over this observation due to a protective effect observed in over 3 year olds [211]. A more recent analysis of asthmatic children, referred to a clinic for respiratory symptoms found that levels of UFPs in breath condensate correlated with wheezing, eosinophilia and breath symptom score [212]. Other studies in children have also indicated some correlation with increased levels of UFPs and functional asthma outcomes but again additional confounding factors cannot be ruled out [213–216].

Additional investigations to identify the effects of NMs on asthma have centred mainly on experimentally controlled exposure studies in human volunteers. These include a study examining the effect of isolated UFPs collected from a motor vehicle polluted area in the US, which found decreases in lung function (FEV_1_). There was, however, no difference between asthmatic and control volunteers [217]. Other studies have investigated the effects of inhaled CNPs as model UFPs. Allergic asthmatic subjects exposed to CNPs 28 days before allergen challenge demonstrated increased markers of disease including eosinophilia. There was however no alteration in lung function (FEV_1_) [218]. Another study found that inhalation of CNPs caused mild small airway dysfunction together with impaired alveolar gas exchange in normal subjects. When examined in asthmatic subjects there were no differences in responses to those observed for normal volunteers [219]. An additional study from the same group found that there were some small changes in vasculature function after CNPs exposure but there were no changes in asthmatic symptoms, pulmonary function, or markers of airway inflammation in both asthmatic and control volunteers [220]. This study did, however, identify that asthmatic subjects have an increased deposition of NMs after inhalation as compared to control volunteers, an observation also found in other studies [221, 222]. This effect raises the possibility of increased potentially hazardous effects of these materials in asthmatic populations due to higher exposures over the long term.

Despite there being a weight of evidence for adverse effects of UFPs on conditions such as cardiovascular disease in humans [223–225], the picture is less clear for asthmatic disease. Much of the epidemiology suffers from a lack of ability to separate the effects of UFPs from other pollutant material, while human volunteer exposure studies are limited and do not offer clear cut evidence for a direct impact. The studies described in this section focus almost completely on the potential for acute exacerbation effects of these nanosized materials. Apart from a study indicating long term and repeated exposure to NP abrasion products from diamond polishing [226] and a case report on long term exposure to Nickel NPs [227], there are virtually no long term epidemiological analysis of the effects of NPs and their potential to influence the development of asthma.

### Experimental models of ultrafine air pollutant exposure effects in asthma

Concern for how particulate pollutants and the nanoscale constituents therein, contribute to adverse health effects were raised by early *in vivo* observations that pulmonary exposure of rats to urban PM10 produces an inflammatory response. In one such study the authors demonstrated a greater inflammatory response with CNPs and proposed that the UFP component of PM10 was the main contributor to the effects seen [228]. In this section we will summarise those studies that have attempted to examine the effect of the UFP fraction of air pollution on models of asthma exacerbation and development (Table 3).

**Table 3:** Ultrafine pollutant particular matter effects in experimental *in vivo* models of asthma.

Nanomaterial Properties			Model Characteristics				Disease Related Endpoints									Ref
*Material*	*1° Size (nm)*	*Location*	*Species (strain)*	*Allergen (route)*	*Exposure Route*	*Dose*	*AHR (test)*	*IgE*	*Eos*	*Neu*	*Lym*	*MΦ*	*Total cell*	*Gene Expression Markers*	*GCH-MS*	
Ultrafine Particles	<150	Los Angeles	Mice (BALB/c -f)	OVA (Int N)	Int N	0.95 μg/kg	-	↑	nc	nc	nc	-	nc	↑ (TNF-α, IL-5, IL-13, IL-6, MCP-1, MIPI-α) nc (IL-4, INF-γ)	-	[231]
DCB-230	200	-	Rats (BN–neonates)	n/a	A	200 μg/m^3^ (20min x7d)	↑ (Resist)	-	nc	↑	↑	-	-	↓ (IL-18, VEGF, TNF-α, MCP-1, IL-6, IL-1β)	-	[234]
Ultrafine Particles	<180	Downtown Los Angeles	Mice (BALB/c –f)	OVA (Int N)	Int N	0.01 μg/kg	-	nc	↑	nc	↑	-	↑	↑ (IL-12, IL-6, IL-1β) nc (IL-13)	-	[232]
Ultrafine Particles	<150	Downtown Los Angeles	Mice (BALB/c –f)	OVA (Int N)	Int N	297 – 394 μg/m3	-	nc	nc	nc	-	-	-	↑ (IL-5) nc (IL-13)	-	[256]
Ultrafine Particles	<170	Fresno, CA, USA (Summer Night)	Mice (BALB/c –m)	HDM (Int N)	Int N	45 μg/m3	-	-	nc	nc	nc	nc	nc	nc (HO-1)	-	[229]
Ultrafine Particles	<170	Fresno, CA, USA (Winter Day)	Mice (BALB/c –m)	HDM (Int N)	Int N	45 μg/m3	-	-	↑	nc	↑	nc	↑	↑ (HO-1)	-	[229]
Ultrafine Particles	<170	Fresno, CA, USA (Winter Night)	Mice (BALB/c –m)	HDM (Int N)	Int N	45 μg/m3	-	-	nc	nc	nc	nc	nc	nc (HO-1)	-	[229]
Ultrafine Particles	<170	Fresno, CA, USA (Summer Day)	Mice (BALB/c –m)	HDM (Int N)	Int N	45 μg/m3	-	-	nc	nc	↑	nc	nc	↑ (HO-1)	-	[229]
Ultrafine Particles	<180	Los Angeles	Mice (BALB/c –f)	OVA (Int N)	Int N	0.04 μg/kg	-	↑	↑	↑	-	-	-	↑ (IL-5, IL-13, IL-17a)	↑ sig MUC5AC	[258]
Ultrafine Particles	<180	Los Angeles	Mice (Il4raR576)	OVA (IP)	Int N	0.18 μg/kg	↑ (Resist)	↑	↑	-	-	-	↑	↑ (IL-4, IL-13, IL-17, IFN-γ, IL-5)	-	[259]
Ultrafine Particles	<180	Detroit (upwind/downwind)	Mice (BALB/c –f)	OVA (IP)	OA	0.28, 1.4 μg/kg	-	nc	nc	nc	-	-	-	↑ (IL-5 (1.4 only))	-	[257]

Abbreviations: 1 primary, *AHR* airway hyperresponsiveness, *LF* lung function, *Resist* Resistance, *Eos* eosinophils, *Neu* neutrophils, *Lym* lymphocytes, MΦ macrophages, *GCH-MS* goblet cell hyperplasia-mucus secretion, −*f* –female, *OVA* ovalbumin, *Int N* intranasal instillation, ↑ increase, *nc* no change; − not determined, *DCB* combustion-generated ultrafine particles containing environmental persistent free radicals using 1,2-dichlorobenzene, *BN* Brown Norway, *A* aerosol, ↓ decrease, − not determined, −*m* –male, *HDM* house dust mite, *IP* intraperitoneal injection, *OA* oropharyngeal aspiration

The UFP fraction of urban PM produced a greater lung inflammatory response than the larger sub-micron-sized fraction, when administered during the sensitisation phase of a HDM murine model of AAD. The effects were correlated with an oxidative stress cellular response [229]. Labile chemicals were suggested as responsible for the increases in antigen presentation marker expression on monocytes induced by coarse, fine and ultrafine fractions of freshly collected urban PM [230]. Indeed, in a murine OVA model of AAD, the higher level of redox cycling organic chemicals, PAHs and intrinsic oxidative potential within the ultrafine fraction (<150nm) of ambient PM, was put forward as the mechanism for increased adjuvancy observed with these particulates when compared to the larger PM2.5 sized fraction [231]. The idea that oxidative potential of ultrafine material is a determining factor for adjuvant behaviour was also supported by another study from the same group, which demonstrated an increase in Th2 inflammation and augmentation of UFP directed adjuvant activity in KO mice lacking the anti-oxidant transcription factor Nrf2 in dendritic cells [232]. Moreover, maternal exposure of mice to free radical chemical containing UFPs (engineered flame combustion particulates) resulted in enhanced OVA induced AAD in offspring, including increased AHR, eosinophils and Th2 responses, which was attributable to an imbalance in T-cell development and inhibition of Th1 maturation [233]. Using similar particles also containing free radical chemicals, the same group demonstrated increased oxidative injury and AHR in mice. These effects were not observed however when UFPs had the pro-oxidant chemicals removed [234].

In another study, these oxidant chemical containing UFPs were observed to induce EMT in infant mouse lungs. The authors suggest that these changes could represent a mechanism through which pollutant material affects airway development and predisposition to conditions such as asthma [235]. In a similar study, UFPs high in PAH chemicals were observed to have increased toxicity in neonatal as compared to adult mice. The authors suggest that neonates have an impaired response to environmental exposures that make them susceptible to pollutant oxidant toxicity and that this may impact airway development and predispose to pulmonary disease development [236]. The effect of nanoscale properties as opposed to adhered chemicals effects in these studies were not investigated, but given that these materials have carbonaceous properties which also have the potential to induce oxidative stress [237] it is possible that the NPs themselves also contribute. Indeed, CNPs as well as OVA and NO_2_ have all been observed to reduce the rate of end expiratory lung volume increase in maturing rats, indicating a direct impact on lung development [238]. As reduced lung function has been put forward as a contributor for the development of asthma [239] and given the observations that air pollution exposure is associated with compromised lung development [240, 241], the potential for both nanoscale effects and chemical components within pollutant materials to adversely affect lung development, and thus ultimately to influence the development asthma is an important consideration that warrants further investigation.

*In vitro* models investigating the effects of ultrafine pollutant material on aspects of asthma pathophysiology have focussed mainly on airway epithelial responses. An early study demonstrated that UFPs (<0.18μm), but not the larger-sized fractions of ambient urban PM, induced GMCSF production from primary human bronchial epithelial cells. This effect on GMCSF, which is a cytokine involved in activation of dendritic cells and Th2 type allergic inflammatory responses was not replicated by exposure of cells to similar sized CNPs, with the suggestion that chemicals adhered to the UFPs were responsible for the observed effects [242]. Fine and UFP fractions from urban Paris were also found to induce GMCSF from the human bronchial 16HBE14o- epithelial cell line [243]. A subsequent study from the same group correlated GMCSF production from airway epithelium with the organic fraction, also suggesting that chemical composition in PM is a major determinant of this response [244]. In a separate study also examining Paris urban as well as rural PM, induction of GMCSF was only induced by the smaller UFP and fine fractions and was correlated to the induction of CYP1A1, NQO1 and HO-1, pointing towards chemical induced aryl hydrocarbon receptor activation (indicative of PAH content) and oxidative stress inducing activity as contributory agents [245]. A more recent study of Lebanese fine and UFPs also indicated the chemical organic fraction as inducing significant effects *in vitro* [246].

Diesel exhaust particulates (DEPs) are estimated to account for a significant proportion of the UFP fraction found in ambient and traffic related air pollution. There is evidence that DEPs levels are associated with exacerbation of pre-existing asthma and more recently evidence also points towards a direct impact on sensitisation and respiratory effects in early life [247, 248]. Indeed, DEPs have been observed to produce adjuvant effects in humans [249]. Experimental modelling in mice further supports the hypothesis that respiratory exposure to DEPs can act as an adjuvant [250]. Similar to studies of ambient PM in mice, it has been proposed that the chemical content, in particular the PAH content of DEPs, are the major contributor to adjuvant activity [251]. In contrast, work carried out by Tanaka and colleagues revealed that exposure of mice to NP rich DEPs, optimised to maintain relevant organic chemical content, resulted in an adjuvant effect on OVA induced AAD. Interestingly, this adjuvant effect remained when particulates were filtered from the exhaust material, implicating gaseous pollutants as the main effector of adjuvancy [252]. The examination of gaseous pollutants, such as NO_2_ and ozone, for adjuvant effects in experimental AAD has revealed mixed responses [253, 254]. Clarity is still needed regarding the relative contribution of individual pollutant components including the UFP fraction, in complex mixtures.

Exacerbation of AAD, including effects on AHR has been documented in experimental models of ambient PM exposure [255]. The extent to which individual size fractions within such material contribute to exacerbation outcomes has also been examined. Exposure of OVA sensitised mice to the PM2.5 fraction collected close to a motorway resulted in an increase in inflammatory responses, which were not observed to the same extent with UFP fraction exposures [256]. Another study examining near road collected PM and exacerbation of inflammatory events in OVA sensitised mice found that the PM10 and PM2.5 fractions resulted in a greater inflammatory cell response than the UFP fraction, with responses more pronounced in samples with a higher level of traffic related material [257]. The UFP fraction of near motorway PM has been observed to exacerbate OVA induced AAD in mice but this study did not examine other fractions of PM to allow size comparisons to be made [258]. Both the fine (PM2.5) and UFP fractions of traffic associated PM were found to exacerbate AAD in OVA sensitised mice through a mechanisms involving JAG1 and the NOTCH signalling pathway, events which were not observed with carbon black fine particle exposure [259]. This study also examined antigen presentation aspects and T-cell responses *in vitro* and *in vivo* and through interventional and knockout strategies demonstrated that UFP induced exacerbation of AAD, including IgE responses was mediated through the aryl hydrocarbon receptor. The authors proposed that since UFPs have chemicals associated with them including PAHs, which are potent ligands for the aryl hydrocarbon receptor, that such receptor activation drives JAG1 expression in dendritic cells and consequently allows enhancement of T-cell responses to aggravate AAD [259].

Additional aspects of exacerbation impacts have been discussed as a consequence of PM and UFP exposure, including inhibition of macrophage phagocytosis and clearance of microorganisms [260]. Interestingly, a recent study demonstrated that neonates exposed to model ultrafine particulates with high oxidant potential enhanced the severity of influenza virus infection [261]. Whether similar effects occur for other respiratory viral infections, including asthma associated rhinovirus and RSV exacerbations, is currently unknown.

## Knowledge gaps and future directions

Broadly speaking from the studies summarised in this review, there appears to be evidence for an impact of NMs on asthma and AAD. There are however many significant knowledge gaps that need to be addressed in order to build any weight of evidence towards a confident assignment of hazard potential relevant for human exposure. Specifically there is an urgent need for much more detailed and mechanistic information surrounding the vast collection of different types of NM and their properties and how they may each affect the range of different manifestations of asthma and AAD observed in humans. There is also a need for study designs to be tailored towards more human relevant exposure scenarios, in order to increase confidence in testing being relevant for human disease. Only a handful of nanomaterial inhalation studies have been carried out to date. These studies capture relevant particle dynamics, properties and deposition patterns, not properly accounted for when the nanomaterial is administered in solution. Future efforts should encourage inhalation exposures coupled with real world dose equivalents to minimise translational uncertainties. Furthermore, there is a lack of information on long term exposures to NMs. New studies together with the development of new approaches, including *in vitro* and mechanistic insight to address these knowledge gaps are to be encouraged.

### Nanomaterial characteristics as a predictor for adverse effects

The application of appropriate NM characterisation approaches is at the forefront of addressing knowledge gaps. This is fundamental to accurately attribute nanoscale properties to biological interaction and toxicological outcome. The extent to which NM characterisation was carried out across the studies in this review can be considered substantially inadequate in the vast majority of cases. Adoption of guidance proposals for minimum sets of NM characterisation requirements previously proposed in regulatory and other broader contexts [262, 263] would greatly enhance the power to assign hazard potential in future work.

How one may confidently assign particular characteristics of NMs to specific adverse outcome endpoints, is a substantial challenge and has only been attempted on a limited scale. Initial attempts were made to compare nanoparticle composition and characteristics across studies in this review. However, it was ultimately judged unworkable as the variabilities in models used; including, exposure protocol, material dose and route, time points and endpoints of disease, among many other parameters, including the extent of NM characterisation, made it impossible to reliably compare. Therefore the only realistic comparisons that can be made to assign NM characteristics to adverse effects with any degree of confidence are from those studies, which focussed on addressing NM characteristics within a specific study. These studies are summarised in Table 4.

**Table 4:** Summary studies of *in vivo* exposures and focussed investigation of engineered nanomaterial characteristics

Material Type	Models of Asthma and Allergic Airway Disease				Nanomaterial Characteristics Specifically Investigated for Effects in Models of Asthma and Allergic Airway Disease							
	Allergen Exposure Models of AAD			Pulmonary Exposure	Primary Size	Solubility	Shape/Structure	Agg size	Charge	Composition	Surface Area	Surface Chemistry/Coating
	ALLERGEN INDEP	SENSITISATION	EXACERBATION									
CNPs	[41] [40] [60] [37] [38] [59] [58] [62] [238]	[37] [38] [39] [40] [41] [42] [43] [54] [72] [238]	[58] [59, 60] [61, 62] [110]	[127] [277]	[37] [38] [39] [40] [42] [54] [40] [37] [38]		[54]			[43] [127]	[54]	
MWCNT	[169] [134] [135] [50] [278] [67] [69] [70] [51] [49]	[49] [50, 51] [54]	[67] [68] [69] [70] [71]	[147]	[49] [54]		[49] [54] [134]				[54]	
SWCNT	[136] [52]	[52, 53] [54]			[54] [52]		[54] [52]				[54]	
CNF		[54]			[54]		[54]				[54]	
AgNP	[132] [111] [116]		[111] [112] [116] [274]		[132]				[132]			[132]
AuNP			[63] [117]							[63]		
Co_3_O_4_NP				[126] [127] [279]						[126] [127] [279]		
CuONP	[64] [64]		[64]	[279] [280]		[280]				[279] [280]		
NiONP				[126] [127] [279] [280] [281] [282] [146]		[280] [281]				[126] [127] [279] [280]		
TiO_2_NP	[119] [133] [81] [119]	[43] [55] [120] [81] [273]	[63] [120] [118] [119] [273]	[126] [127] [283]		[55]				[43] [55] [63] [126] [127]		
CeO_2_NP				[104]								
ZnONP	[56] [56]	[55] [56]		[280] [194]		[55] [56] [280] [194]				[55] [280]		[55]
Fullerene		[106]										
FeNP	[275] [80]	[80]	[275] [93]									
GONP	[65]	[65]	[65]									
Latex NP	[272]	[272]			[272] [272]							
PSPNP	[79]	[44] [79]			[44]							
SiO_2_NP	[48] [46] [47] [276] [48]	[45] [46, 47] [48] [55]	[66] [276]		[45] [55]	[55]		[48]	[66]	[55]	[48] [55] [276]	[48] [66] [276]
TOTALS	42	30	27	12	17	5	4	1	2	7	4	5

Abbreviations: *Agg* agglomerate, *GO* Graphene Oxide, *MWCNT* multi-walled carbon nanotubes, *SWCNT* Single-walled carbon nanotubes, *CNF* Carbon Nanofibres

Size dependent effects have been observed for sensitisation, inflammation and airway disease parameters. This cannot however be said to be a universal effect for all materials and disease situations (due to lack of ability to compare across studies). Interestingly, smaller-sized NMs such as AgNPs and ZnONPs were observed to have more of an impact on mast cells and eosinophils *in vitro* when compared to larger sized particles of the same material. In addition, smaller primary-sized particles of both AuNPs and AgNPs materials were associated with inhibitory effects on AAD, while larger-sized particles were associated with exacerbation effects. While it is interesting to speculate as to whether there is a common size dependent mechanism involved here, the observations are too few and the models used too variable to reliably say. Importantly, no *in vivo* studies have reported the role of nanomaterial size on exacerbation effects in AAD. This is a significant knowledge gap in our understanding.

Agglomeration size whether in aerosol or instillation fluid was not documented to the same extent as primary NP size. Agglomeration size and its potential to influence disease is an often overlooked and little discussed characteristic that may influence tissue distribution and cellular interaction. In addition, how the transition to and from agglomerate state(s) affects biological interaction and localisation (including subcellular) and whether this behaviour is material and/or nano property dependent is unknown.

Solubility, surface charge and surface coating of NPs have been a focus for a number of studies and have demonstrated potential mechanisms and concerns. However these too would be considered of insufficient weight to attribute hazard with any great confidence to any specific property or material. The nature of the biological corona surrounding NPs as they interact with living tissue has been put forward as an important factor determining toxicological impact [264, 265]. To what degree this is true and to what extent material type and associated properties influences this, remains to be determined.

In terms of NP composition, attempts can be made at some general observations, albeit with obvious caveats. CNTs would appear to have a strong adjuvant activity and a detrimental impact on AAD when exposure occurs during sensitisation. In contrast, the effect of CNTs on exacerbation is absent or present to a lesser extent. In some studies, CNTs have been associated with an increase in Th2 type inflammation in the absence of adaptive immune responses, indicating a possible concern for exacerbation of intrinsic type asthma. Whether such categorisation based on asthma endotype can be made confidently, is possibly premature but is something that should be considered in the future. In addition, whether these observations can be linked to the high aspect ratio of CNTs is unknown but also worth further investigation. Some materials like titanium and fullerenes are associated more with inhibitory effects on AAD than others. In addition, there are a number of materials that have not been tested in different types of exposure settings. For example AuNPs, AgNPs and CuONPs have all been tested for their ability to affect pre-existing AAD but not for any effect on sensitisation and the development of AAD. SWCNTs on the other hand have been examined for adjuvant effects but not investigated for any impact on pre-existing disease.

Surface area is a critical factor for pulmonary toxicity of NMs, particularly soluble metal NPs [266]. Much more work is needed to investigate this NM characteristic as a determinant of adverse effects of NMs in asthma and AAD. Concerted efforts to categorise NMs according to their adverse effects by reference to their characteristics such as size, solubility, surface reactivity, shape (e.g. high aspect ratio, spherical, fibres, platelet-like) and metal content among other characteristics is a particular focus for nanotoxicology. While the lack of NM characterisation data, together with model variability, in the studies in this review currently limits attempts to link adverse effects to these groupings, it is imperative to prioritise such approaches for future work.

### Model selection, comparability across studies and protocol standardisation

**Fig. 1:**
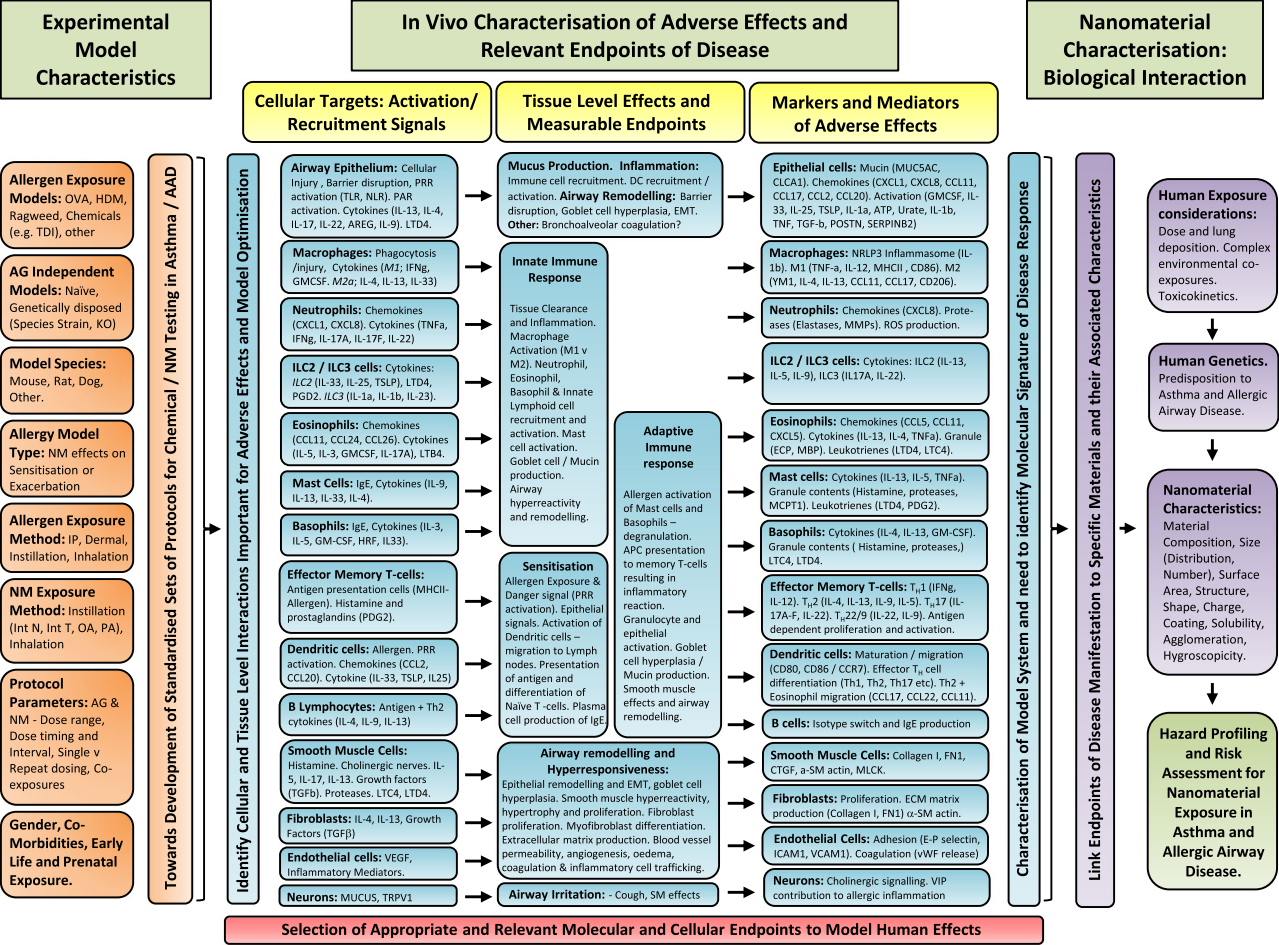
Investigation of Hazard Potential of NM in Experimental In Vivo Models of Asthma and AAD: Key Events and Endpoints of Disease

There is a large variability in the types of protocol and disease-associated endpoints used to assess adverse effects relevant for asthma and AAD, which make it extremely difficult to directly compare results across studies. Adoption of a standardised set of protocols, addressing these concerns would allow more confident comparisons to be made across studies. We have summarised those approaches used to date in terms of model set-up and characteristics, target cells and tissues, key events in disease manifestation as well as molecular endpoints of adverse effects (Fig. 1) in order to highlight the immense set of challenges, which need to be addressed if one is to bring forward NM testing approaches to become more informative and relevant for human disease.

Given the diversity of asthma in terms of atopic, non-atopic and mixed forms of the disease together with the different stages of susceptibility (e.g. sensitisation and exacerbation), the ability to compare a defined set of disease indicators specific for the type of disease stage, across studies would aid greatly in further clarifying NM effects within a standardised protocol framework. Importantly, reversible airway obstruction is the primary observation used to diagnose those with asthma, and AHR assessment is used to model this *in vivo*. There is a significant knowledge gap surrounding the effects of NMs on AHR as well as a lack of examination of smooth muscle effects and airway remodelling as underlying causes and targets. This can also be said for mucin production and goblet cell hyperplasia modelling as another overlooked and underreported aspect to airway disease in asthma and NM exposure. Both of these features should be included as a basic requirement for any NM studies examining the potential impact on asthma and AAD.

There is also an overrepresentation of *in vivo* modelling using ovalbumin sensitisation protocols. The inclusion of more human relevant antigen exposures such as house dust mite may allow for broader capture of effects that may otherwise be protocol type specific. Aerosolised delivery of nanomaterials in test systems is also significantly lacking in studies to date. This is particularly important given that this is the route by which humans are exposed to NM. The consequences that instillation of NM dispersions have on deposition patterns, nanomaterial characteristics and biological response is rarely discussed. Moreover, the range of doses applied across all studies is highly variable and how it relates to human exposure levels is not often referred to.

### Translational relevance for human nanomaterial and pollutant exposure

Information on direct effects of engineered nanomaterials on asthma and AAD in humans is limited and evidence for the potential for adverse effects comes mainly from *in vivo* and *in vitro* experimental modelling. However, inadvertent exposure to airborne PM mainly from traffic related pollution brings with it the concern that nanosized particles contained within this inhaled mixture have particular detrimental health effects including an influence on asthma. Epidemiological studies have suggested a role for the UFP fraction of pollutant material in asthma exacerbation, although the role for confounding factors including other pollutants cannot be entirely excluded. Evidence from experimental modelling also suggests that the UFP fraction from ambient and traffic related pollutant material may play a role in the sensitisation and development of AAD. However, experimental models *in vivo* suggest that the UFP fraction may be less important for exacerbation effects than the larger fractions, particularly the fine (typically PM1 and PM2.5) fractions. This apparent discordance with epidemiological observations necessitates clarification.

Such discussion of ultrafine effects in Asthma and AAD must also be considered in the context of other constituents of air pollutant materials. Across all experimental studies including *in vitro* work, there is strong evidence that the particle associated chemical component, containing PAHs among other organics, rather than the particles themselves are the critical determinant responsible for pollutant particulate mediated biological impact in asthma and AAD. However, given that nanomaterials including carbon nanoparticles can directly impact these same model systems, further investigation is needed to address direct versus indirect particulate effects within a pollutant mixture. It should not be overlooked that more labile and gaseous components of airborne pollution also are likely to have a significant role [252].

Population studies have pointed towards genetic susceptibility for air pollutant exposures and asthma outcome. These include exposures such as PM10, PM2.5, ozone and NO_2_ and effects associated with altered asthma outcomes in those with particular single nucleotide polymorphisms in genes such as GSTP1, NQO1 and GSTT1 [267–269]. These genes are part of the anti-oxidant defence pathway within the cell, altered function of which may impact susceptible individuals through an inability to mount an appropriate anti-oxidant response. However, gene-environment interactions have not been investigated for associations with the smaller UFP fraction of airborne pollution and represent a significant knowledge gap. This is also true to a degree for engineered NP exposure, although some initial studies comparing different animal strains suggest genetic background is a strong determinant of pulmonary response to inhaled or instilled NMs [117, 119, 132]. Indeed, a wider analysis of up to 20 different strains of mice for responses to inhaled ZnONPs found that there were up to 10 fold differences in PMN accumulation in the lung [270]. Similarly, in a study of 8 different mouse strains, lung inflammation and injury in response to inhaled AgNPs was also found to be strain dependent [271]. These later studies did not focus on models of asthma or AAD but do highlight pulmonary response susceptible populations. Interestingly, no studies to date have been reported examining how genetic background affects NM impact on sensitisation and development of AAD. Other types of susceptibilities that exist and may play a role in how pollutant UFPs and engineered NMs impact asthma include *in utero* exposure, age, gender, diet and co-morbidities, including obesity, and have not been examined to any great extent.

### Mechanistic understanding and focus for future testing

**Fig. 2:**
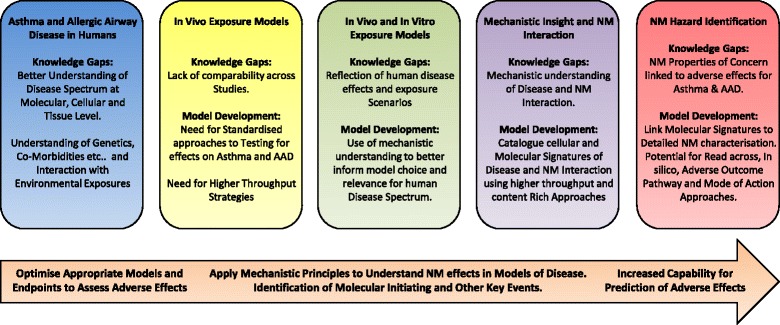
Approaches for the Development of a Better Understanding and Prediction of Adverse Effects of NM on Asthma and AAD

The development of new and more refined approaches to NM testing for effects on asthma and AAD have begun to incorporate concepts of how mechanistic information can lead to more detailed understanding of the most important molecular targets responsible for pathophysiological change. Identification of molecular initiating events that are coupled to detailed mechanistic understanding and NM characterisation, furthers the potential to more clearly define NM properties of concern. A more complete understanding of events that are responsible for NM effects at a tissue, cellular and molecular level through the use of standardised *in vivo* protocols can also lead to the development of more targeted testing approaches such as those using *in vitro* modelling. While *in vitro* modelling to date has been used to identify adverse effects as they may affect sensitisation and other approaches, these remain abstracted from other cellular and tissue compartments that may have important contribution to the overall NM effect at an organism level. Through greater understanding of *in vivo* targets we can begin to prioritise and standardise *in vitro* tests, which may ultimately replace animal testing. The adoption of *in vitro* systems has many advantages including the ability to greatly increase the number of different NMs that can be tested. Given the many combinations of different material types and characteristics, this is crucial. *In vitro* testing will also allow for testing to be carried in a human rather than an animal system, removing particular concerns regarding species specific effects (Fig. 2). In addition, technologies including the use of induced pluripotent stem cells (iPSC) to develop *in vitro* testing models will allow for the integration of population diverse as well as human disease specific genetic backgrounds, further optimising towards human relevant testing. Of particular note is the recently funded Marie Curie - Innovative Training Network “in3”, which aims to incorporate iPSC models into an overall strategy to develop better models for chemical and NM safety assessment.

The recent momentum behind understanding modes of toxicity and development of adverse outcome pathways for xenobiotic effects has the potential to help translate *in vitro* findings to a whole organism level. Additionally, it is the hope that through accrual of data from standardised and higher throughput systems, that *in silico* modelling and read across strategies may develop to a point where experimental testing for new materials can be minimised (Fig. 2). Through a greater and more detailed understanding of how NMs may impact respiratory disease outcome and the identification of adverse effect linked molecular signatures, including epigenetic marks, it is hoped that more informative biomarkers of NM exposure and effect may be developed. These may also be useful for refining epidemiological studies of asthma and NM or air pollutant exposure.

### Microbial exposure

Lastly, it is becoming ever clearer that microbial exposure is a critical factor influencing both the development and exacerbation of asthma and AAD. Exposure to bacterial and fungal diversity within the microbiome has been strongly associated with a reduced development of atopy and asthma [8]. Viral infection is also considered a major factor for both the development and exacerbation of disease. These interactions and their biological effects should not be examined in isolation from other environmental exposures such as air pollution. Whether NMs can influence these microbial interactions remains to be fully explored.

## Conclusions

Within this review we have produced a detailed analysis of studies directed to investigate how nanomaterials including nano-sized pollutant particulates may impact asthma and allergic airway disease. We have identified significant knowledge gaps including a lack of investigations targeted towards understanding how different nanomaterial properties influence disease outcome *in vivo*. Attempts at standardisation for testing may overcome the diverse nature of different disease models and allow meaningful comparison of different nanomaterials and their effects across studies. The development of *in vitro* models as representative of *in vivo* exposure and disease progression is immature, particularly in terms of how nanomaterial effects on airway remodelling may be captured. This is also true for how multiple cell types may interact to produce adverse effects. In terms of human exposure, epidemiological studies are rare for engineered materials and represent another important knowledge gap. Investigations into how pollutant nanomaterials affect asthma and AAD in human populations are far from clear due to the inability to separate co-pollutant effects.

In order to address these knowledge gaps, mechanistic understanding at a cellular and molecular level, of disease pathology as well as NM specific outcomes is required. Some attempts have been made in studies covered in this review. Apart from some direct effects on the process of sensitisation involving dendritic cells and studies examing specific gene function through KO approaches, mechanistic insight is generally lacking. As knowledge progresses in our understanding of human asthma development and exacerbation events, it is important that cellular and molecular discoveries underlying adverse effects and outcomes are incorporated into model development. This allows for a more targeted and translational approach to testing. With the inclusion of more detailed NM characterisation, this approach also allows for increased power to identify properties of concern that can be linked more readily to relevant endpoints of disease. Specifically, in terms of adjuvant activity and the development of sensitisation to antigen, there is a need to determine how the innate immune response, including airway epithelial, macrophage, mast and dendritic cells handle and respond to different NMs as they are first encountered within the lung. How NM properties including size and surface area impact how antigens attached to their surface are handled by these cells is something to consider as a priority research area. For established AAD, there is also a need to tie down how NMs propagate an allergic response. There is also a need for models to be able to capture information relevant for other asthma endotypes such as intrinsic asthma or severe asthma, where allergy does not play a role. Mechanistic insight should be prominent in our attempts to achieve this. Finally, an understanding of airway remodelling and hyper-reactivity is urgently needed; given these events underlie airway obstruction, arguably the pathological feature with the greatest impact on patient morbidity and mortality. Mechanistic understanding of physical airway responses and in particular how smooth muscle cells, fibroblasts and other airway resident cells react to NM exposure is lacking and should also be encouraged as a priority research area.

## Additional file

Additional file 1: Table S1. Effects of nanomaterials in non-allergic *in vivo* models of experimental pulmonary exposure. Table S2. Allergen independent effects of nanomaterials in *in vivo* models of experimental pulmonary exposure documented as part of larger allergy driven models of asthma and allergic airway disease. Table S3. *In vitro* models and nanomaterial exposure. (DOCX 295 kb)
