# Supplementary material for: Mechanistic insight into the impact of nanomaterials on asthma and allergic airway disease
Source: Part Fibre Toxicol. 2017 Nov 21;14:45. doi: 10.1186/s12989-017-0228-y (PMC5697410; doi:10.1186/s12989-017-0228-y)
Supplement: Additional file 1: Table S1. — Effects of nanomaterials in non-allergic in vivo models of experimental pulmonary exposure. Table S2. Allergen independent effects of nanomaterials in in vivo models of experimental pulmonary exposure documented as part of larger allergy driven models of asthma and allergic airway disease. Table S3. In vitro models and nanomaterial exposure. (DOCX 295 kb) [file 12989_2017_228_MOESM1_ESM.docx]

**Supplementary Table 1.** Effects of nanomaterials in non-allergic *in vivo* models of experimental pulmonary exposure.

| Nanomaterial Properties | | | | Model Characteristics | | | | |  | | Disease Related Endpoints | | | | | | | |  |
| --- | --- | --- | --- | --- | --- | --- | --- | --- | --- | --- | --- | --- | --- | --- | --- | --- | --- | --- | --- |
| *Material (coating)* | ***Size (nm)*** | ***Surface area (m^2^/g)*** | ***Agg***  ***size***  ***(nm)*** | ***Species (Strain - Gender)*** | ***Particle (Route)*** | ***Dose*** | ***AHR***  ***(LF test)*** | ***IgE*** | | ***Eos*** | | ***Neu*** | ***Lym*** | ***MΦ*** | ***Total Cells*** | ***Pathophysiological Indicators*** | ***GCH-MS*** | ***Reference*** | |
| Ag  (citrate) | 24 | - | - | Rat  (BN-m) | Int T | 0.1  mg/kg | 🡩  (Resist) | 🡩 | | nc | | 🡩 | nc | nc | 🡩 | 🡩 (IL-13, CCL11) nc (IL-5) | - | [[1](#_ENREF_1)] | |
| Ag  (citrate) | 24 | - | - | Rat  (SD –m) | Int T | 0.1  mg/kg | nc (Resist) | nc | | 🡩 | | 🡩 | 🡩 | 🡩 | 🡩 | nc (IL-13, CCL11, IL-5) | - | [[1](#_ENREF_1)] | |
| Ag  (PVP) | 26 | - | - | Rat  (BN-m) | Int T | 0.1  mg/kg | 🡩  (Resist) | 🡩 | | nc | | 🡩 | nc | nc | 🡩 | 🡩 (IL-13, CCL11 IL-5) | - | [[1](#_ENREF_1)] | |
| Ag  (PVP) | 26 | - | - | Rat  (SD –m) | Int T | 0.1  mg/kg | nc (Resist) | nc | | nc | | nc | nc | nc | nc | nc (IL-13, CCL11 IL-5) | - | [[1](#_ENREF_1)] | |
| C | 14 | 253.9 | - | Rat  (Wistar –m) | Int T | 6.9 mg/kg | - | - | | nc | | 🡩 | nc | 🡫 | nc | 🡩 (MIP-2) | - | [[2](#_ENREF_2)] | |
| Co_3_O_4_ | 20 | 36.9 | - | Rat  (Wistar –m) | Int T | 6.9 mg/kg | - | - | | nc | | 🡩 | nc | 🡫 | nc | 🡩 (MIP-2) | - | [[2](#_ENREF_2)] | |
| Ni | 20 | 36.2 | - | Rat  (Wistar –m) | Int T | 6.9 mg/kg | - | - | | nc | | 🡩 | nc | 🡫 | nc | nc (MIP-2) | - | [[2](#_ENREF_2)] | |
| TiO_2_ | 20 | 49.8 | - | Rat  (Wistar –m) | Int T | 6.9 mg/kg | - | - | | nc | | nc | nc | nc | nc | nc (MIP-2) | - | [[2](#_ENREF_2)] | |
| C | 14 | 300 | - | Mice  (JF1/Msf –f) | Int T | 3 mg/kg | - | - | | - | | 🡩 | nc | 🡩 | - | 🡩 (IL-1β, TNF-α, IL-4, CCL20) | - | [[3](#_ENREF_3)] | |
| Co_3_O_4_ | 18.4 | 35.8 | 185.2 | Rat  (Wistar –f) | Int T | 1.9 mg/kg | - | - | | - | | 🡩 | 🡩 | 🡩 | 🡩 | 🡩 (MCP-1) nc (IL-12, INF-γ, IL-17A, IL-4) | - | [[4](#_ENREF_4)] | |
| CuO | 23.1 | 29 | 112.1 | Rat  (Wistar –f) | Int T | 2.2 mg/kg | - | - | | - | | 🡩 | nc | nc | 🡩 | 🡩 (MCP-1, IL-12, IL-17α) nc (INF-γ, IL-4) | - | [[4](#_ENREF_4)] | |
| NiO | 5.3 | 91.8 | 92.5 | Rat  (Wistar –f) | Int T | 0.7 mg/kg | - | - | | - | | 🡩 | 🡩 | 🡩 | 🡩 | 🡩 (MCP-1) nc (IL-12, IL-17A, INF-γ, IL-4) | - | [[4](#_ENREF_4)] | |
| Co_3_O_4_ | 20 | 47.9 | - | Rat  (Wistar –m) | Int T | 5.2 mg/kg | - | - | | - | | 🡩 | nc | 🡫 | 🡩 | - | - | [[5](#_ENREF_5)] | |
| Ni | 20 | 43.8 | - | Rat  (Wistar –m) | Int T | 5.2 mg/kg | - | - | | - | | 🡩 | nc | 🡫 | 🡩 | - | - | [[5](#_ENREF_5)] | |
| TiO_2_ | 28 | 45 | - | Rat  (Wistar –m) | Int T | 5.2 mg/kg | - | - | | - | | 🡩 | nc | 🡫 | 🡩 | - | - | [[5](#_ENREF_5)] | |
| NiO | 20 | 104.6 | <100 | Rats  (Wistar –m) | Int T | 0.66  mg/kg | - | - | | - | | - | - | 🡩 | - | 🡫 (IL-2) 🡩 (MIP-1α)  nc (IL-1β, IL-4, IL-5) | - | [[6](#_ENREF_6)] | |
| Ni | ~20 | 40-60 | - | Mice  (WT –m) | OA | 4  mg/kg | - | - | | nc | | 🡩 | nc | 🡫 | nc | 🡩 (CCL2) | nc  PAS | [[7](#_ENREF_7)] | |
| NiO | 5.3 | 91.8 | 224 | Rats  (Wistar –f) | Int T | 1.6  mg/kg | - | nc | | 🡩 | | 🡩 | - | 🡩 | 🡩 | nc (IL-1β, CCL11) | - | [[8](#_ENREF_8)] | |
| TiO_2_ | 21 | - | 100 | Rat  (F-344 –b) | A | 12mg/ m^3^ x5.6h/dx3 | 🡩 (Resist) | - | | - | | - | - | - | nc | 🡩 (ROS) nc (IL17, IL-4, IL1β, MCP1, TNFα) | nc | [[9](#_ENREF_9)] | |
| MWCNT (rigid) | D >50, L 13μm | - | - | Mice  (C57BL/6 -f) | A | 7.2mg/m^3^ x4h x4d | 🡩 (Penh) | - | | 🡩 | | 🡩 | 🡩 | 🡫 | - | 🡩 (IL-13, IL-5) 🡫 (INF-γ) | 🡩 PAS | [[10](#_ENREF_10)] | |
| MWCNT (tangled) | D >50, L 10-50μm | - | - | Mice  (C57BL/6 -f) | A | 18mg/m^3^ x4h x4d | - | - | | nc | | nc | nc | nc | - | nc (IL-13, IL-5, IFN-γ) | nc PAS- | [[10](#_ENREF_10)] | |
| MWCNT | D 22.5, L 10-100μm | 113.10 | - | Mice (C57BL/6) | OA | 4  mg/kg | 🡩 (Resist) | - | | - | | 🡩 | - | - | - | 🡩 (IL-33)  nc (IL-6) | - | [[11](#_ENREF_11)] | |
| MWCNT | D 27, L-5-15μm | - | - | Mice (C57B1/6) | OA | 2.7  mg/kg | 🡩 (Resist) | - | | 🡩 | | 🡩 | - | 🡫 | 🡩 | 🡩 (IL-33, IL-5, IL-6, CCL11) | - | [[12](#_ENREF_12)] | |
| CeO_2_ | 8 | 44 | 90 | Mice (C57B1/6-m) | OA | 2.7  mg/kg x1 | - | - | | - | | 🡩 | 🡩 | 🡩 | 🡩 | 🡩 (IL-6, MIP1a) | - | [[13](#_ENREF_13)] | |
| MWCNT | D 10-20 | - | 262 | Mice (C57B1/6-f) | OA | 1.5  mg/kg x1 | - | - | | 🡩 | | 🡩 | - | 🡩 | - | 🡩 (IL1β, KC, TNFα) | - | [[14](#_ENREF_14)] | |
| ZnO | 9 | 88 | 66.9 | Mice (C3H/NeH) | A | 0.86 mg/m3 | - | - | | - | | 🡩 | - | 🡩 | - | 🡩 (MCP-1, CXCL1) | - | [[15](#_ENREF_15)] | |
| SWCNT | D 2-10, L-0.3-6μm | 480 | - | Mice  (ICR-m) | Int T | 5.5-27  mg/kg x1 | 🡩 (Resist) | - | | nc | | 🡩 | 🡩 | 🡩 | 🡩 | 🡩 (MCP-1, MIP1a) | - | [[16](#_ENREF_16)] | |
| TiO_2_ | 21 | - | 200-2000 | Rats (Dark Agouti -m) | Int T | 5mg/kg x1 | - | - | | 🡩 | | 🡩 | 🡩 | nc | 🡩 | 🡩 (IL1β, IL-1α, IL-6, IL-4, IL-10) | - | [[17](#_ENREF_17)] | |
| NiO | 10-20 | 91.8 | 92.5 | Rats  (Wistar –f) | Int T | 0.65  mg/kg x1 | - | - | | nc | | 🡩 | 🡩 | - | 🡩 | 🡩 (IL1β, MIP-2) | - | [[18](#_ENREF_18)] | |
| ZnO | <10 | 48.2 | 422.5 | Rats  (Wistar –f) | Int T | 1.25  mg/kg x1 | - | - | | 🡩 | | 🡩 | nc | - | 🡩 | 🡩 (IL1β) | - | [[18](#_ENREF_18)] | |
| Cu | <50 | 29 | 112.1 | Rats  (Wistar –f) | Int T | 2.1  mg/kg x1 | - | - | | 🡩 | | 🡩 | nc | - | 🡩 | 🡩 (IL1β, MIP-2) | - | [[18](#_ENREF_18)] | |

**Abbreviations:** Agg, agglomerate; AHR, airway hyperresponsiveness; LF, lung function; Resist, Resistance; Eos, eosinophils; Neu, neutrophils; Lym, lymphocytes; MΦ, macrophages; GCH-MS, goblet cell hyperplasia-mucus secretion; BN, Brown Norway; -m, -male; Int T, intratracheal instillation; ↑, increase; nc, no change; - , not determined; SD, Sprague Dawley; PVP, Poly Vinyl Pyrrolidone; ↓, decrease; -f, -female; WT, Wild Type; OA, oropharyngeal aspiration; PAS, Periodic acid–Schiff staining; MWCNT, multi-walled carbon nanotubes; D, diameter; L, length; A, aerosol; SWCNT, single-walled carbon nanotubes; DA, Dark Agouti.

**Supplementary Table 2.** Allergen independent effects of nanomaterials in *in vivo* models of experimental pulmonary exposure documented as part of larger allergy driven models of asthma and allergic airway disease.

| Nanomaterial Properties | | | | Model Characteristics | | |  | Disease Related Endpoints | | | | | | | |
| --- | --- | --- | --- | --- | --- | --- | --- | --- | --- | --- | --- | --- | --- | --- | --- |
| *Material (coating)* | ***Size (nm)*** | ***Surface area (m^2^/g)*** | ***Agg size (nm)*** | ***Species (Strain - Gender)*** | ***Particle (Route)*** | ***Dose*** | ***AHR***  ***(LF test)*** | ***IgE*** | ***Eos*** | ***Neu*** | ***Lym*** | ***Total cells*** | ***Pathophysiological Indicators*** | ***GCH-MS*** | ***Reference*** |
| Ag | 6 | - | - | Mice  (C57BL/6 –f) | A | 40mg/kg x5d | nc  (Penh) | - | nc | nc | nc | nc | 🡫 (IL-4)  nc (IL-5, IL-13) | - | [[19](#_ENREF_19)] |
| Ag | 33 | - | - | Mice  (BALB/c –f) | A | 3.3mg/m^3^ x6h x7d | 🡩  (Penh) | nc | nc | nc | nc | - | nc (IL-13)  🡩 (LTE4) | - | [[20](#_ENREF_20)] |
| CuO | 46.67 | - | 375.8 | Mice  (BALB/c –f) | Int N | 100  μg/kg | 🡩  (Penh) | 🡩 | - | 🡩 | 🡩 | 🡩 | 🡩 (ROS, IL-1β, IL-6, TNF-α) | 🡩 PAS | [[21](#_ENREF_21)] |
| C | 14 | 300 | - | Mice  (ICR –m) | Int T | 2.8 mg/kg | nc (Resist) | - | - | - | - | - | - | nc MUC5AC | [[22](#_ENREF_22)] |
| C | 14 | - | 887 | Mice  (BALB/c –f) | PA | 2.5  mg/kg | - | - | nc | nc | nc | nc | 🡩 (CXCL1, IL-4, IL-13) | - | [[23](#_ENREF_23)] |
| C | 20 | 442 | - | Mice  (C57BL/6 –f) | PA | 10  mg/kg | - | - | - | 🡩 | - | 🡩 | - | - | [[24](#_ENREF_24)] |
| C | 14 | 300 | - | Mice  (ICR –m) | Int T | 1.61  mg/kg x6 | - | nc | nc | 🡩 | - | 🡩 | 🡩 (MCP-1)  nc (IL-5, IL-13, IL-4, IL-6) | nc Goblet cells | [[25](#_ENREF_25)] |
| C | 56 | 45 | - | Mice  (ICR –m) | Int T | 1.61  mg/kg x6 | - | nc | nc | 🡩 | - | 🡩 | nc (IL-5, IL-13, IL-4, IL-6, MCP-1) | nc Goblet cells | [[25](#_ENREF_25)] |
| C | 14 | 300 | - | Mice  (ICR –m) | Int T | 1.61  mg/kg x6 | - | - | - | - | - | - | 🡩 (MIP-1α, TARC)  nc (IL-2, IL-10) | - | [[26](#_ENREF_26)] |
| C | 56 | 45 | - | Mice  (ICR –m) | Int T | 1.61  mg/kg x6 | - | - | - | - | - | - | 🡩 (MIP-1α, TARC)  nc (IL-2, IL-10) | - | [[26](#_ENREF_26)] |
| C | 7 | - | - | Mice  (BALB/c –f) | A | 507  μg/m^3^ | - | - | nc | nc | - | - | nc (LTB_4_, 8-isoprostane, PGE_2_) | - | [[27](#_ENREF_27)] |
| C | 56 | 45 | - | Mice  (ICR –m) | Int T | 1.61  mg/kg x6 | nc (Resist) | - | - | - |  | - | - | nc MUC5AC | [[22](#_ENREF_22)] |
| C | 35.2 | - | - | Dog  (Beagle) | A | 232.3  μg/m^3^ | nc (Resist) | nc | nc | 🡩 | nc | nc | - | - | [[28](#_ENREF_28)] |
| C | 7 | - | - | Mice  (BALB/c) | A | 507μg/m^3^  x24h | - | - | - | - | - | - | 🡩 (TNF-α, CC-16) | - | [[29](#_ENREF_29)] |
| C | 13 | - | <10 μm | Rat  (BN –m) | Int T | 0.5  mg/kg x3 | nc  (Resist) | nc | nc | 🡩 | nc | nc | nc (IFN-γ, IL-1β, TNF-α, IL-4, IL-5, IL-13) | - | [[30](#_ENREF_30)] |
| Α-Fe_2_O_3_ | 30 | 28.98 | 950-2000 | Mice  (BALB/c –f) | Int T | 5  mg/kg | - | nc | 🡩 | 🡩 | 🡩 | 🡩 | nc (TNF-α, Il-1β, IL-6) | - | [[31](#_ENREF_31)] |
| Fe_2_O_3_ | 35 | 39 | - | Mice  (BALB/c –f) | Int T | 27.7  mg/kg x4 | - | nc | nc | nc | nc | - | nc IL-4, INF-γ | - | [[32](#_ENREF_32)] |
| Graphene | D 0.61, L 0.20-2μm | - | - | Mice  (BALB/c –f) | PA | 4.5  mg/kg | - | nc | nc | nc | nc | - | nc (IL-4, IL-5, IL-13) | nc Goblet cells | [[33](#_ENREF_33)] |
| Latex | 25 | - | - | Mice  (ICR –m) | Int T | 8 mg/kg x6 | - | nc | nc | 🡩 | 🡩 | 🡩 | 🡩 (IL-18, MIP-1α) nc (IL-1β, MCP-1, IL-5, IL-13) |  | [[34](#_ENREF_34)] |
| Latex | 50 | - | - | Mice  (ICR –m) | Int T | 8 mg/kg x6 | - | nc | nc | 🡩 | 🡩 | 🡩 | 🡩 (MCP-1 IL-18, MIP-1α)  nc (IL-1β, IL-5, IL-13) |  | [[34](#_ENREF_34)] |
| Latex | 100 | - | - | Mice  (ICR –m) | Int T | 8 mg/kg x6 | - | nc | nc | nc | nc | nc | 🡩 (MCP-1, IL-18, MIP-1α)  nc (IL-1β, IL-5, IL-13) |  | [[34](#_ENREF_34)] |
| Polystyrene | 50 | - | - | Mice  (BALB/c –f) | Int T | 12.5 mg/kg | - | - | 🡩 | 🡩 | - | 🡩 | 🡩 (IL-6, IL-12, GMCSF, MCP-1) 🡫 (IL-5, IL-13) | - | [[35](#_ENREF_35)] |
| SiO_2_ | 10-20 | 140-180 | - | Rat  (Wistar –m) | Int I | 0.48mg/kg x30 | nc (Resist) | - | nc | - | - | - | 🡩 (IL-4)  nc (INF-γ) | - | [[36](#_ENREF_36)] |
| SiO_2_  (PEG) | 90 | - | - | Mice  (BALB/c –f) | Int N | 22.2  mg/kg | - | nc | nc | 🡩 | nc | - | 🡩 (MIP-1α) nc (IL-6, IL-4, IL-5, IL13,TNF-α,INF-γ) | nc PAS | [[37](#_ENREF_37)] |
| SiO_2_ | ~100 | 12.7 | - | Mice  (BALB/c –f) | Int N | 11 mg/kg x3 | 🡩 (Resist) | - | nc | nc | 🡩 | 🡩 | nc (IL-5, IL-13, IL-1β, INF-γ) | - | [[38](#_ENREF_38)] |
| SiO_2_  (PEG) | ~100 | 12.7 | - | Mice  (BALB/c –f) | Int N | 11 mg/kg x3 | nc (Resist) | - | nc | nc | nc | nc | nc (IL-5, IL-13, IL-1β, INF-γ) | - | [[38](#_ENREF_38)] |
| SiO_2_  (Meso) | 100 | 70.6 | - | Mice  (BALB/c –f) | Int N | 11 mg/kg x3 | nc (Resist) | - | nc | nc | nc | nc | nc (IL-5, IL-13, IL-1β, INF-γ) | - | [[38](#_ENREF_38)] |
| SiO_2_ | 100 | 12.7 | 119.6 | Mice  (BALB/c –f) | Int N | 10 mg/kg x6 | nc (Resist) | - | - | 🡩 | nc | 🡩 | 🡩 (IL-5) nc (IL-13, IL-1β, INF-γ) | nc PAS | [[39](#_ENREF_39)] |
| SiO_2_ (Meso) | 100 | 70.6 | 100.5 | Mice  (BALB/c –f) | Int N | 10 mg/kg x6 | 🡩 (Resist) | - | - | 🡩 | nc | 🡩 | 🡩 (IL-5, IL-13, INF-γ) nc (IL-1β) | nc PAS | [[39](#_ENREF_39)] |
| SiO_2_ (PEG) | 100 | 12.7 | 439.1 | Mice  (BALB/c –f) | Int N | 10 mg/kg x6 | nc (Resist) | - | - | 🡩 | nc | 🡩 | nc (IL-5, IL-13, IL-1β, INF-γ) | nc PAS | [[39](#_ENREF_39)] |
| TiO_2_  (SiO_2_) | 10x40 | 132 | - | Mice  (BALB/c –f) | A | 10mg/m^3^ 2h x12d | - | nc | nc | 🡩 | nc | - | 🡩 (CXCL5) nc (IL-1β, TNF-α, IL-4, IL-13, IL-10) | - | [[40](#_ENREF_40)] |
| TiO_2_ | 21 | - | 4200 | Rat  (DA-m) | A | 159 μg, deposited | 🡩 (Resistance) | - | - | 🡩 | 🡩 | 🡩 | 🡩 (IL-1α, IL-1β,IL-6, IL-7, INF-γ, TNF-α, CCL20)  nc (IL-5, IL-17, IL-2) | nc PAS | [[41](#_ENREF_41)] |
| TiO_2_ | 21 | - | 4200 | Rat  (BN –m) | A | 168 μg, deposited | nc (Resist) | - | - | 🡩 | 🡩 | 🡩 | 🡩 (IL-1α, IL-1β, TNF-α)  nc (IL-6, INF-γ, IL-7, IL-5, IL-17, IL-2, CCL20) | nc PAS | [[41](#_ENREF_41)] |
| ZnO | 50 | 10.8 | - | Mice  (Balb/c –f) | OA | 0.5  mg/kg | - | nc | 🡩 | 🡩 | - | 🡩 | 🡩 (IFN-ɣ, TNF-α IL-4, IL-5, IL-6, IL-13) | - | [[42](#_ENREF_42)] |
|  |  |  |  |  |  |  |  |  |  |  |  |  |  |  |  |
| MWCNT | D 10-15 L 1-10μm | - | - | Mice  (BALB/c –m) | Int N | 1 mg/kg | nc (Resist) | nc | nc | 🡩 | nc | 🡩 | 🡩 (IL-13)  nc (IL-4, IL-5, IL-17) | - | [[43](#_ENREF_43)] |
| MWCNT | D 10-30 L 0.5-40μm | 12.83 | - | Mice  (ICR–m) | Int T | 50  mg/kg | - | nc | - | 🡩 | nc | 🡩 | 🡩 (IL1, IL10, IL-12, TNF-α) nc (IL-4, IL-5, INF-γ) | - | [[44](#_ENREF_44)] |
| MWCNT | D 30-50 L 0.3-50μm | 109.29 | - | Mice  (C57BL/6 –m) | A | 100  mg/m^3^ | - | - | nc | 🡩 | nc | - | 🡩 (MCP-1)  nc (IL-13, IL-5, TGF-β1) | - | [[45](#_ENREF_45)] |
| MWCNT | D 49, L 3.56μm | - | - | Mice  (WT –m) | OA | 4 mg/kg | - | - | nc | 🡩 | nc | nc | 🡩 (IL-1β, TNF- α, TGF-β)  nc (IL-13, IL-10) | - | [[46](#_ENREF_46)] |
| MWCNT | D 30-50 L 0.3-50μm | 109.29 | - | Mice  (C57BL/6J) | OA | 4  mg/kg | - | nc | nc | nc | nc | - | nc (IL-13, IL-5, IL-17α, CXCL10, TGFβ1) | - | [[47](#_ENREF_47)] |
| MWCNT | D 10-15 L 0.1-10μm | 200–500 | - | Mice  (BALB/c –m) | Int N | 12 mg/kg x3 | - | - | nc | 🡩 | nc | nc | nc (TGF-β1, IL-13, IL-33)  🡩 (TSLP, IL-25) | 🡩 PAS | [[48](#_ENREF_48)] |
| MWCNT | D 67, L 0.1-10μm | 26 | - | Mice  (ICR –m) | Int T | 4.6 mg/kg x6 | - | nc | nc | 🡩 | 🡩 | 🡩 | nc (IL-4) 🡫 (IL-5) 🡩 (IL-13, INF-γ, IL-18, IL-33, MCP-1, IL-1β) | - | [[49](#_ENREF_49)] |
| SWCNT | D 1.2-2  L 1-15µm | - | - | Mice  (ICR –m) | Int T | 1.3 mg/kg x7 |  | nc | nc | 🡩 | 🡩 | 🡩 | - | nc PAS | [[50](#_ENREF_50)] |
| SWCNT | D 0.8-1.2  L 0.1-1µm | - | - | Mice  (ICR –m) | Int T | 1.3 mg/kg x7 | - | nc | nc | 🡩 | 🡩 | 🡩 | 🡩 (IL-33, INF-γ, IL-17, IL-23, MCP-1)  nc (IL-4, IL-5, IL-13) | nc PAS | [[50](#_ENREF_50)] |

**Abbreviations:** Agg, agglomerate; AHR, airway hyperresponsiveness; LF, lung function; Resist, Resistance; Eos, eosinophils; Neu, neutrophils; Lym, lymphocytes; GCH-MS, goblet cell hyperplasia-mucus secretion; BN, Brown Norway; -m, -male; Int T, intratracheal instillation; ↑, increase; nc, no change; - , not determined; SD, Sprague Dawley; PVP, Poly Vinyl Pyrrolidone; ↓, decrease; -f, -female; WT, Wild Type; OA, oropharyngeal aspiration; PAS, Periodic acid–Schiff staining; MWCNT, multi-walled carbon nanotubes; D, diameter; L, length; A, aerosol; SWCNT, single-walled carbon nanotubes; DA, Dark Agouti.

**Supplementary Table 3.** *In vitro* models and nanomaterial exposure.

| Nanomaterial Properties | | | | Model Characteristics | | | | Disease Related Endpoints |  |
| --- | --- | --- | --- | --- | --- | --- | --- | --- | --- |
| *Material (Coating)* | ***Size (nm)*** | ***Surface area (m^2^/g)*** | ***Agg***  ***size***  ***(nm)*** | ***Cell Type*** | ***Dose (μg/ml)*** | ***Exposure Length*** | ***Culture type*** | ***Pathophysiological Indicators*** | **Reference** |
| C | 22 | 341 | <220 | Splenic leukocytes (T-cells) | 12  + OVA | 72h | Suspension | 🡫stat4  🡩 IL-4, IL-13, IL-10 | [[51](#_ENREF_51)] |
| C | 39 | 54.4 | <220 | Splenic leukocytes (T-cells) | 12  + OVA | 72h | Suspension | 🡫stat4 downregulated  🡩 IL-4 | [[51](#_ENREF_51)] |
| Fullerene (Poly-OH) | - | - | - | Peripheral Blood Basophils | 0.1 | 30m-30h | Suspension | 🡫 (Degranulation. IL-13, ROS) | [[52](#_ENREF_52)] |
| Fullerene (Poly-OH) | - | - | - | Lung mast cells | 0.1 | 30m-30h | Suspension | 🡫 (Degranulation. PGD2), | [[52](#_ENREF_52)] |
| C | 10-50 |  | 200-500 | RAW267.9 Macrophages | 0.005 - 10 | 48h | Submerged | 🡩 Cytotoxicity  nc IL-12, IL-10 | [[53](#_ENREF_53)] |
| MWCNT | D 5-30, L 30nm-3 μm | - | 1-3 μm | RAW267.9 Macrophages | 0.005 - 10 | 48h | Submerged | 🡩 Cytotoxicity  nc IL-12, IL-10 | [[53](#_ENREF_53)] |
| MWCNT | D 10-30, L 50nm-1μm | - | 0.1-3 μm | RAW267.9 Macrophages | 0.005 – 10 | 48h | Submerged | 🡩 Cytotoxicity  nc IL-12, IL-10 | [[53](#_ENREF_53)] |
| SWCNT | D 10-50 | - | 2-20 μm | RAW267.9 Macrophages | 0.005 – 10 | 48h | Submerged | 🡩 Cytotoxicity  nc IL-12, IL-10 | [[53](#_ENREF_53)] |
| MWCNT | D – 10-30  L- 0.5-40μm | 40-300 | 129 | Primary Human Bronchial Epithelial | 1.5 - 24 | 16-48h | Submerged | 🡩 IL-1β, IL-18, IL-8.  NLRP3 | [[54](#_ENREF_54)] |
| MWCNT | D 20-30,  L 5-10μm | - | 324 | THP-1 (human monocyte) | 5-100 | 24h | Suspension | 🡩 IL-1β, STAT6 | [[55](#_ENREF_55)] |
| TiO_2_ | ~50nm | 36 | - | ChaGo-K1 | 0.05-1mg/ml | 15mins | Submerged | 🡩 mucin production (all doses) | [[56](#_ENREF_56)] |
| Au  (Bet v 1) | 53.1 | - | - | Human Primary Basophils | 0.657 | - | Suspension | nc (Basophil activation; compared to non-conjugated allergen) | [[57](#_ENREF_57)] |
| Au  (Der p 1) | 54.2 | - | - | Human Primary Basophils | 0.578 | - | Suspension | 🡩 (Basophil activation; compared to non-conjugated allergen) | [[57](#_ENREF_57)] |
| Au  (Phl p 5) | 54.6 | - | - | Human Primary Basophils | 0.365 | - | Suspension | nc (Basophil activation; compared to non-conjugated allergen) | [[57](#_ENREF_57)] |
| SWCNT | D 1.2-2  L 1-15µm | - | - | Murine bone marrow derived dendritic cells | 0.1-5 | 24-96h | Submerged | 🡩 (CD86, T-cell reactivity, NO) | [[50](#_ENREF_50)] |
| C | 14 | - | 887 | Murine bone marrow derived dendritic cells | 0.3-30 | 12h | Submerged | nc (CD86, CCR7) | [[23](#_ENREF_23)] |
| C | 14 | - | 887 | Murine bone marrow derived dendritic cells | 0.3-30  BAL CM | 12h | Submerged | 🡩 (CD86, CCR7) | [[23](#_ENREF_23)] |
| C | 30-50 | - | - | Murine bone marrow derived dendritic cells | 25 | 24h | Submerged | 🡩 (CD86, CD80, CD40) | [[58](#_ENREF_58)] |
| TiO_2_ | 60 | - | - | Rat RBL-2H3 mast cells | 100-750 | 5m | Suspension | 🡩 (Histamine release) | [[59](#_ENREF_59)] |
| SiO2 | 70 | - | - | Mouse DC2.4 dendritic cell line | 2.9–15 | 24h | Submerged | 🡩 (T-cell Cross presentation – IL-2 lease from CD8-OVA1.3 cells.) | [[60](#_ENREF_60)] |
| SiO2 (Amine) | 70 | - | - | Mouse DC2.4 dendritic cell line | 2.9–15 | 24h | Submerged | nc (T-cell Cross presentation – IL-2 lease from CD8-OVA1.3 cells.) | [[60](#_ENREF_60)] |
| SiO2 (Carboxyl) | 70 | - | - | Mouse DC2.4 dendritic cell line | 2.9–15 | 24h | Submerged | nc (T-cell Cross presentation – IL-2 lease from CD8-OVA1.3 cells.) | [[60](#_ENREF_60)] |
| SiO2 (PEG-Carboxyl) | 51 | - | - | OT-I CD8+ T-cells | 10 | 48h | Suspension | 🡩 (INF-γ, IL-2) | [[61](#_ENREF_61)] |
| SiO2 (PEG-Carboxyl) | 51 | - | - | OT-II CD4+ T-cells | 10 | 48h | Suspension | nc (INF-γ, IL-2) | [[61](#_ENREF_61)] |
| Airborne Particulates | <100 | - | - | Blood derived Monocytes | 50 | 24h | Submerged | 🡩 (HLA-DR, CD40, CD80, CD86) | [[62](#_ENREF_62)] |
| SiO2 | 30 | - | - | Murine bone marrow derived dendritic cells | 10 μg/cm2 | 24h | Submerged | 🡩 (IL-1β, IL-18) | [[63](#_ENREF_63)] |
| Magnetic Iron (PVA) | 29 | - | - | Monocyte derived Dendritic cells | 10-20 | 12h-5d | Submerged | 🡫 (Antigen processing, T-cell activation, IL-1β, IL-5, INF-γ, IL-12p70) 🡩 (IL-10) | [[64](#_ENREF_64)] |
| SiO_2_ | 14 | 200 | - | Murine bone marrow derived dendritic cells | 20-50 μg/cm2 | 18h | Submerged | 🡩 (CD86, CD80, MHC II) | [[65](#_ENREF_65)] |
| TiO_2_ | 20-80 | 50 | - | Murine bone marrow derived dendritic cells | 20-50 μg/cm2 | 18h | Submerged | 🡩 (CD86, CD80, MHC II) | [[65](#_ENREF_65)] |
| TiO_2_ | 7-10 | 239 | 25 | Human monocyte derived Dendritic cells | 1.7 | 24-5d | Submerged | 🡩 (CD83, CD86, CD80, CCR7, IL-12, TNFa); T-cells 🡩 (INF-γ, TNFa), nc (IL-4, IL-5, IL-10) | [[66](#_ENREF_66)] |
| CeO_2_ | 3-5 | 90 | 10 | Human monocyte derived Dendritic cells | 1.7 | 24-5d | Submerged | nc (CD83, CD86, CD80, CCR7, IL-12, TNFa); T-cells nc (INF-γ, TNFa), 🡩 (IL-4, IL-5, IL-10) | [[66](#_ENREF_66)] |
| C | 14 | 300 | - | Murine bone marrow derived dendritic cells | 5-25 | 24-3d | Submerged | 🡩 (DEC205, CD86, T-cell activation) | [[67](#_ENREF_67)] |
| C | 56 | 45 | - | Murine bone marrow dendritic cells | 5-25 | 24-3d | Submerged | 🡩 (DEC205, CD86, T-cell activation) | [[67](#_ENREF_67)] |
| TiO_2_ | 29 | - | - | Human monocyte derived Dendritic cells | 10 | 24h | Submerged | nc (MHC II, CD40, CD80, CD83, CD86, CD209) | [[68](#_ENREF_68)] |
| ZnO | 17 | - | - | Human monocyte derived Dendritic cells | 10 | 24h | Submerged | nc (MHC II, CD40, CD80, CD83, CD86, CD209) | [[68](#_ENREF_68)] |
| Au | 10 | - | - | Murine bone marrow derived dendritic cells | 98.5 | 24h | Submerged | nc (MHC II, IL-6); 🡩 (IL-12p70) | [[69](#_ENREF_69)] |
| CuO | <50 | - | - | Human airway epithelial NCI-H292 cell line | 2 | 24h | Submerged | 🡩 (MUC5AC, IL-6, IL-8) | [[70](#_ENREF_70)] |
| SWCNT | D 10-21. L 2-10uM. | - | 0.1-1 uM | Rat RBL-2H3 mast cells | 10-100 | 24h | Suspension | 🡫 (Mast cell activation) | [[71](#_ENREF_71)] |
| Ag (PVP) | 21 | - | - | Murine bone marrow derived mast cells | 6.25-50 | 1h | Suspension | 🡩 (Mast cell degranulation - b-hexosaminidase release) | [[72](#_ENREF_72)] |
| Ag (Citrate) | 18 | - | - | Murine bone marrow derived mast cells | 6.25-50 | 1h | Suspension | 🡩 (Mast cell degranulation - b-hexosaminidase release) | [[72](#_ENREF_72)] |
| TiO_2_ | 1-10 | - | 87 | Human peripheral blood derived neutrophils | 100 | 30mins | Suspension | 🡩 degranulation (MPO, MMP9 release) | [[73](#_ENREF_73)] |
| CeO_2_ | 1-10 | - | 61 | Human peripheral blood derived neutrophils | 100 | 30mins | Suspension | 🡩 degranulation (MPO, MMP9 release) | [[73](#_ENREF_73)] |
| ZnO | 1-10 | - | 10-56 | Human peripheral blood derived neutrophils | 100 | 30mins | Suspension | 🡩 degranulation (MPO, MMP9 release) | [[73](#_ENREF_73)] |
| C | 90 | 750 | - | Human primary bronchial epithelial cells | 33 | 24h | Submerged | 🡩 IL-8 | [[74](#_ENREF_74)] |
| ZnO | 21 | - | - | Rat RBL-2H3 mast cells | 5-20 | 20mins | Suspension | 🡫 Mast cell degranulation (β-hexosaminidase release) | [[75](#_ENREF_75)] |
| Ag | 8-47 | - | 68 | HL-60 Neutrophil cell line | 3.9-15.6 | 2mins – 24h | Suspension | 🡩 activation (intracellular Ca2+, SO2-). 🡩 (IL-8, MCP1) | [[76](#_ENREF_76)] |
| C | 13 | 350 | - | Human bronchial epithelial 16HBE14o cells | 10 | 24h | Submerged | 🡩 (GMCSF, IL-6, TNF –α) | [[77](#_ENREF_77)] |
| TiO_2_ | 15 | 190-290 | - | Human bronchial epithelial 16HBE14o cells | 10 | 24h | Submerged | 🡩 (GMCSF, IL-6, TNF –α) | [[77](#_ENREF_77)] |
| MWCNT | D 32nm. L 0.07-7.8uM | 106.7 | 5700 | Human airway epithelial BEAS2B cells | 5-40 | 24h | Submerged | 🡩 (IL-8). nc (IL-6, TNF –α) | [[78](#_ENREF_78)] |
| MWCNT (-OH) | D 18nm. L 0.02-1.7uM | 129.8 | 3030 | Human airway epithelial BEAS2B cells | 5-40 | 24h | Submerged | nc (IL-8, IL-6, TNF –α) | [[78](#_ENREF_78)] |
| MWCNT (-COOH) | D 24nm. L 0.03-1.5uM | 139.1 | 5100 | Human airway epithelial BEAS2B cells | 5-40 | 24h | Submerged | nc (IL-8, IL-6, TNF –α) | [[78](#_ENREF_78)] |
| CeO_2_ | 15 | 61 | >1000 | Human 3D reconstituted airway epithelium (MucilAir) | 30-333 ug/cm2 | 3h | ALI | 🡩 (IL-8, MCP-1) | [[79](#_ENREF_79)] |
| MWCNT | D 10-20 | - | 262 | Human primary bronchial epithelial cells | 25-50 | 24h | Submerged | 🡩 (IL-8, NLRP3) | [[14](#_ENREF_14)] |
| MWCNT | D 10-20 | - | 262 | Human 3D reconstituted airway epithelium (MucilAir-HF) | 25-50 | 24h | ALI | 🡩 (IL-8, , IL-1β,  TNF –α, GM-CSF, ROS) | [[14](#_ENREF_14)] |
| MWCNT | D 10-20 | - | 262 | Human Primary alveolar macrophages | 25-50 | 24h | Submerged | nc (IL-1β, TNF –α) | [[14](#_ENREF_14)] |
| SiO_2_ | 27.5 | 65 | 77 | Human airway epithelial BEAS2B cells | 50-200 | 20h | Submerged | 🡩 (IL-8, IL-6) | [[80](#_ENREF_80)] |
| CoO | 7 | - | - | Human airway epithelial BEAS2B cells | 6.1 | 6-24h | Submerged | 🡩 (CCL28) | [[81](#_ENREF_81)] |
| CeO_2_ | 4 | - | - | Human airway epithelial BEAS2B cells | 1.27 | 6-24h | Submerged | nc (CCL28) | [[81](#_ENREF_81)] |
| ZnO | 9 | 88 | 156 | Human airway epithelial BEAS2B cells | 100uM | 48h | Submerged | 🡩 (IL-8) | [[15](#_ENREF_15)] |
| CuO | 5-9 | - | 17 | Human primary bronchial epithelial cells | 83-166ng / well | 2-4h | ALI | 🡩 (IL-8) | [[82](#_ENREF_82)] |
| SWCNT | D 1nm. L 0.1-1uM | 400-1000 | - | Human airway epithelial BEAS2B cells | 6.6 | 24h | Submerged | 🡩 (IL-8, IL-6, IL-1β, IL-1α) | [[83](#_ENREF_83)] |
| SiO_2_ | 41 | 63 | 1023 | Human airway epithelial BEAS2B cells | 12.5 - 200 | 2h | Submerged | 🡩 (IL-8, IL-6, IL-1β, TNFα, GM-CSF) | [[84](#_ENREF_84)] |
| SiO_2_ | 64 | 65 | 682 | Human airway epithelial BEAS2B cells | 12.5 - 200 | 2h | Submerged | 🡩 (IL-8, IL-6, IL-1β, TNFα, GM-CSF) | [[84](#_ENREF_84)] |
| ZnO | 24–70 | - | - | Human airway epithelial BEAS2B cells | 2-8 | 4-6h | Submerged | 🡩 (IL-8) | [[85](#_ENREF_85)] |
| ZnO | 24–70 | - | - | Human primary bronchial epithelial cells | 8 | 4h | ALI | 🡩 (IL-8) | [[85](#_ENREF_85)] |
| SWCNT | - | - | - | Human primary bronchial epithelial cells | 100 | 48h | Submerged | 🡩 (IL1RL1) | [[86](#_ENREF_86)] |
| CB | 13 | 350 | 253 | Human airway epithelial (16HBE14o-) cells | 5ug/cm2 | 24h | Submerged | 🡩 (GM-CSF) | [[87](#_ENREF_87)] |
| TiO_2_ | 15 | 200-220 | 86-356 | Human airway epithelial (16HBE14o-) cells | 5ug/cm2 | 24h | Submerged | 🡩 (GM-CSF) | [[87](#_ENREF_87)] |
| CeO_2_ | 8 | 44 | 90 | Murine bone marrow derived dendritic cells | 10-100 | 24h | Suspension | 🡩 (OPN, PGD2), nc (IL-6, IL-13, degranulation) | [[13](#_ENREF_13)] |

**Abbreviations:** Agg, agglomerate; ↑, increase; ↓, decrease; nc, no change; - , not determined; SD, Sprague Dawley; PVP, Poly Vinyl Pyrrolidone; MWCNT, multi-walled carbon nanotubes; D, diameter; L, length; SWCNT, single-walled carbon nanotubes.

**Pubmed Search Strings used to capture relevant literature.**

1. Human and In vivo studies.

(asthma OR asthmatic OR asthmagen OR asthmogen OR allergic OR atopic OR allergenic OR allergy OR allergies OR allergen OR allergens OR atopy) AND (nanomaterials OR nanomaterial OR nanoparticle OR nanoparticles OR ultrafine OR ultrafines OR nanotube OR nanotubes OR fullerene OR fullerenes OR nanofiber OR nanofibers OR nanofibre OR nanofibres OR nanoscale OR nanoscience OR nanotechnology OR nanotechnologies OR nanotubes OR nanowire OR nanowires OR nano OR nanoparticulate OR nanoparticulates OR nanosheet OR nanosheets OR nanocube OR nanocubes OR "quantum dot" OR "quantum dots" OR nanorods OR “graphene sheet”)

1. In vivo and In vitro models and exposures.

(dendritic[title] OR lymphocyte[title] OR lymphocytes[title] OR ((Bronchial[title] OR airway[title]) AND epithelial[title]) OR Mast[title] OR “T-cell” [title] OR “B-cell” [title] OR Eosinophil[title] OR basophil[title] OR neutrophil[title] OR Eosinophils[title] OR basophils[title] OR neutrophils[title]OR “smooth muscle” [title]) AND (nanomaterials OR nanomaterial OR nanoparticle OR nanoparticles OR ultrafine OR ultrafines OR nanotube OR nanotubes OR fullerene OR fullerenes OR nanofiber OR nanofibers OR nanofibre OR nanofibres OR nanoscale OR nanoscience OR nanotechnology OR nanotechnologies OR nanotubes OR nanowire OR nanowires OR nano OR nanoparticulate OR nanoparticulates OR nanosheet OR nanosheets OR nanocube OR nanocubes OR "quantum dot" OR "quantum dots" OR nanorods OR “graphene sheet”)

**References**

1. Seiffert J, Hussain F, Wiegman C, Li F, Bey L, Baker W, et al. Pulmonary toxicity of instilled silver nanoparticles: influence of size, coating and rat strain. PloS one. 2015;10 3:e0119726; doi: 10.1371/journal.pone.0119726. <http://www.ncbi.nlm.nih.gov/pubmed/25747867>.

2. Dick CA, Brown DM, Donaldson K, Stone V. The role of free radicals in the toxic and inflammatory effects of four different ultrafine particle types. Inhalation toxicology. 2003;15 1:39-52; doi: 10.1080/08958370304454. <http://www.ncbi.nlm.nih.gov/pubmed/12476359>.

3. Ganguly K, Upadhyay S, Irmler M, Takenaka S, Pukelsheim K, Beckers J, et al. Impaired resolution of inflammatory response in the lungs of JF1/Msf mice following carbon nanoparticle instillation. Respiratory research. 2011;12:94; doi: 10.1186/1465-9921-12-94. <http://www.ncbi.nlm.nih.gov/pubmed/21756372>.

4. Cho WS, Duffin R, Bradley M, Megson IL, Macnee W, Howie SE, et al. NiO and Co3O4 nanoparticles induce lung DTH-like responses and alveolar lipoproteinosis. The European respiratory journal. 2012;39 3:546-57; doi: 10.1183/09031936.00047111. <http://www.ncbi.nlm.nih.gov/pubmed/21828028>.

5. Zhang Q, Kusaka Y, Sato K, Nakakuki K, Kohyama N, Donaldson K. Differences in the extent of inflammation caused by intratracheal exposure to three ultrafine metals: role of free radicals. Journal of toxicology and environmental health Part A. 1998;53 6:423-38. <http://www.ncbi.nlm.nih.gov/pubmed/9537280>.

6. Morimoto Y, Ogami A, Todoroki M, Yamamoto M, Murakami M, Hirohashi M, et al. Expression of inflammation-related cytokines following intratracheal instillation of nickel oxide nanoparticles. Nanotoxicology. 2010;4 2:161-76; doi: 10.3109/17435390903518479. <http://www.ncbi.nlm.nih.gov/pubmed/20795893>.

7. Glista-Baker EE, Taylor AJ, Sayers BC, Thompson EA, Bonner JC. Nickel nanoparticles cause exaggerated lung and airway remodeling in mice lacking the T-box transcription factor, TBX21 (T-bet). Particle and fibre toxicology. 2014;11:7; doi: 10.1186/1743-8977-11-7. <http://www.ncbi.nlm.nih.gov/pubmed/24499286>.

8. Lee S, Hwang SH, Jeong J, Han Y, Kim SH, Lee DK, et al. Nickel oxide nanoparticles can recruit eosinophils in the lungs of rats by the direct release of intracellular eotaxin. Particle and fibre toxicology. 2016;13 1:30; doi: 10.1186/s12989-016-0142-8. <http://www.ncbi.nlm.nih.gov/pubmed/27283431>.

9. Scuri M, Chen BT, Castranova V, Reynolds JS, Johnson VJ, Samsell L, et al. Effects of titanium dioxide nanoparticle exposure on neuroimmune responses in rat airways. Journal of toxicology and environmental health Part A. 2010;73 20:1353-69; doi: 10.1080/15287394.2010.497436. <http://www.ncbi.nlm.nih.gov/pubmed/20818535>.

10. Rydman EM, Ilves M, Koivisto AJ, Kinaret PA, Fortino V, Savinko TS, et al. Inhalation of rod-like carbon nanotubes causes unconventional allergic airway inflammation. Particle and fibre toxicology. 2014;11:48; doi: 10.1186/s12989-014-0048-2. <http://www.ncbi.nlm.nih.gov/pubmed/25318534>.

11. Katwa P, Wang X, Urankar RN, Podila R, Hilderbrand SC, Fick RB, et al. A carbon nanotube toxicity paradigm driven by mast cells and the IL-(3)(3)/ST(2) axis. Small. 2012;8 18:2904-12; doi: 10.1002/smll.201200873. <http://www.ncbi.nlm.nih.gov/pubmed/22777948>.

12. Beamer CA, Girtsman TA, Seaver BP, Finsaas KJ, Migliaccio CT, Perry VK, et al. IL-33 mediates multi-walled carbon nanotube (MWCNT)-induced airway hyper-reactivity via the mobilization of innate helper cells in the lung. Nanotoxicology. 2013;7 6:1070-81; doi: 10.3109/17435390.2012.702230. <http://www.ncbi.nlm.nih.gov/pubmed/22686327>.

13. Wingard CJ, Walters DM, Cathey BL, Hilderbrand SC, Katwa P, Lin S, et al. Mast cells contribute to altered vascular reactivity and ischemia-reperfusion injury following cerium oxide nanoparticle instillation. Nanotoxicology. 2011;5 4:531-45; doi: 10.3109/17435390.2010.530004. <http://www.ncbi.nlm.nih.gov/pubmed/21043986>.

14. Hussain S, Ji Z, Taylor AJ, DeGraff LM, George M, Tucker CJ, et al. Multiwalled Carbon Nanotube Functionalization with High Molecular Weight Hyaluronan Significantly Reduces Pulmonary Injury. ACS nano. 2016;10 8:7675-88; doi: 10.1021/acsnano.6b03013. <http://www.ncbi.nlm.nih.gov/pubmed/27459049>.

15. Chen JK, Ho CC, Chang H, Lin JF, Yang CS, Tsai MH, et al. Particulate nature of inhaled zinc oxide nanoparticles determines systemic effects and mechanisms of pulmonary inflammation in mice. Nanotoxicology. 2015;9 1:43-53; doi: 10.3109/17435390.2014.886740. <http://www.ncbi.nlm.nih.gov/pubmed/24559390>.

16. Hsieh WY, Chou CC, Ho CC, Yu SL, Chen HY, Chou HY, et al. Single-walled carbon nanotubes induce airway hyperreactivity and parenchymal injury in mice. American journal of respiratory cell and molecular biology. 2012;46 2:257-67; doi: 10.1165/rcmb.2011-0010OC. <http://www.ncbi.nlm.nih.gov/pubmed/21960547>.

17. Gustafsson A, Lindstedt E, Elfsmark LS, Bucht A. Lung exposure of titanium dioxide nanoparticles induces innate immune activation and long-lasting lymphocyte response in the Dark Agouti rat. Journal of immunotoxicology. 2011;8 2:111-21; doi: 10.3109/1547691X.2010.546382. <http://www.ncbi.nlm.nih.gov/pubmed/21309687>.

18. Cho WS, Duffin R, Poland CA, Duschl A, Oostingh GJ, Macnee W, et al. Differential pro-inflammatory effects of metal oxide nanoparticles and their soluble ions in vitro and in vivo; zinc and copper nanoparticles, but not their ions, recruit eosinophils to the lungs. Nanotoxicology. 2012;6 1:22-35; doi: 10.3109/17435390.2011.552810. <http://www.ncbi.nlm.nih.gov/pubmed/21332300>.

19. Park HS, Kim KH, Jang S, Park JW, Cha HR, Lee JE, et al. Attenuation of allergic airway inflammation and hyperresponsiveness in a murine model of asthma by silver nanoparticles. Int J Nanomedicine. 2010;5:505-15. <http://www.ncbi.nlm.nih.gov/pubmed/20957173>.

20. Chuang HC, Hsiao TC, Wu CK, Chang HH, Lee CH, Chang CC, et al. Allergenicity and toxicology of inhaled silver nanoparticles in allergen-provocation mice models. International journal of nanomedicine. 2013;8:4495-506; doi: 10.2147/IJN.S52239. <http://www.ncbi.nlm.nih.gov/pubmed/24285922>.

21. Park JW, Lee IC, Shin NR, Jeon CM, Kwon OK, Ko JW, et al. Copper oxide nanoparticles aggravate airway inflammation and mucus production in asthmatic mice via MAPK signaling. Nanotoxicology. 2016;10 4:445-52; doi: 10.3109/17435390.2015.1078851. <http://www.ncbi.nlm.nih.gov/pubmed/26472121>.

22. Inoue K, Takano H, Yanagisawa R, Sakurai M, Abe S, Yoshino S, et al. Effects of nanoparticles on lung physiology in the presence or absence of antigen. International journal of immunopathology and pharmacology. 2007;20 4:737-44. <http://www.ncbi.nlm.nih.gov/pubmed/18179746>.

23. Kroker M, Sydlik U, Autengruber A, Cavelius C, Weighardt H, Kraegeloh A, et al. Preventing carbon nanoparticle-induced lung inflammation reduces antigen-specific sensitization and subsequent allergic reactions in a mouse model. Particle and fibre toxicology. 2015;12:20; doi: 10.1186/s12989-015-0093-5. <http://www.ncbi.nlm.nih.gov/pubmed/26141115>.

24. Unfried K, Kroker M, Autengruber A, Gotic M, Sydlik U. The compatible solute ectoine reduces the exacerbating effect of environmental model particles on the immune response of the airways. Journal of allergy. 2014;2014:708458; doi: 10.1155/2014/708458. <http://www.ncbi.nlm.nih.gov/pubmed/24822073>.

25. Inoue K, Takano H, Yanagisawa R, Sakurai M, Ichinose T, Sadakane K, et al. Effects of nano particles on antigen-related airway inflammation in mice. Respir Res. 2005;6:106; doi: 10.1186/1465-9921-6-106. <http://www.ncbi.nlm.nih.gov/pubmed/16164761>.

26. Inoue K, Takano H, Yanagisawa R, Ichinose T, Sakurai M, Yoshikawa T. Effects of nano particles on cytokine expression in murine lung in the absence or presence of allergen. Archives of toxicology. 2006;80 9:614-9; doi: 10.1007/s00204-006-0075-3. <http://www.ncbi.nlm.nih.gov/pubmed/16482471>.

27. Beck-Speier I, Karg E, Behrendt H, Stoeger T, Alessandrini F. Ultrafine particles affect the balance of endogenous pro- and anti-inflammatory lipid mediators in the lung: in-vitro and in-vivo studies. Particle and fibre toxicology. 2012;9:27; doi: 10.1186/1743-8977-9-27. <http://www.ncbi.nlm.nih.gov/pubmed/22809365>.

28. Barrett EG, Rudolph K, Bowen LE, Muggenburg BA, Bice DE. Effect of inhaled ultrafine carbon particles on the allergic airway response in ragweed-sensitized dogs. Inhalation toxicology. 2003;15 2:151-65; doi: 10.1080/08958370304474. <http://www.ncbi.nlm.nih.gov/pubmed/12528044>.

29. Alessandrini F, Weichenmeier I, van Miert E, Takenaka S, Karg E, Blume C, et al. Effects of ultrafine particles-induced oxidative stress on Clara cells in allergic lung inflammation. Particle and fibre toxicology. 2010;7:11; doi: 10.1186/1743-8977-7-11. <http://www.ncbi.nlm.nih.gov/pubmed/20420656>.

30. Layachi S, Rogerieux F, Robidel F, Lacroix G, Bayat S. Effect of combined nitrogen dioxide and carbon nanoparticle exposure on lung function during ovalbumin sensitization in Brown Norway rat. PloS one. 2012;7 9:e45687; doi: 10.1371/journal.pone.0045687. <http://www.ncbi.nlm.nih.gov/pubmed/23029182>.

31. Gustafsson A, Bergstrom U, Agren L, Osterlund L, Sandstrom T, Bucht A. Differential cellular responses in healthy mice and in mice with established airway inflammation when exposed to hematite nanoparticles. Toxicology and applied pharmacology. 2015;288 1:1-11; doi: 10.1016/j.taap.2015.07.001. <http://www.ncbi.nlm.nih.gov/pubmed/26163175>.

32. Ban M, Langonne I, Huguet N, Guichard Y, Goutet M. Iron oxide particles modulate the ovalbumin-induced Th2 immune response in mice. Toxicology letters. 2013;216 1:31-9; doi: 10.1016/j.toxlet.2012.11.003. <http://www.ncbi.nlm.nih.gov/pubmed/23147377>.

33. Shurin MR, Yanamala N, Kisin ER, Tkach AV, Shurin GV, Murray AR, et al. Graphene oxide attenuates Th2-type immune responses, but augments airway remodeling and hyperresponsiveness in a murine model of asthma. ACS nano. 2014;8 6:5585-99; doi: 10.1021/nn406454u. <http://www.ncbi.nlm.nih.gov/pubmed/24847914>.

34. Inoue K, Takano H, Yanagisawa R, Koike E, Shimada A. Size effects of latex nanomaterials on lung inflammation in mice. Toxicology and applied pharmacology. 2009;234 1:68-76; doi: 10.1016/j.taap.2008.09.012. <http://www.ncbi.nlm.nih.gov/pubmed/18938192>.

35. Hardy CL, Lemasurier JS, Mohamud R, Yao J, Xiang SD, Rolland JM, et al. Differential uptake of nanoparticles and microparticles by pulmonary APC subsets induces discrete immunological imprints. Journal of immunology. 2013;191 10:5278-90; doi: 10.4049/jimmunol.1203131. <http://www.ncbi.nlm.nih.gov/pubmed/24123688>.

36. Han B, Guo J, Abrahaley T, Qin L, Wang L, Zheng Y, et al. Adverse effect of nano-silicon dioxide on lung function of rats with or without ovalbumin immunization. PloS one. 2011;6 2:e17236; doi: 10.1371/journal.pone.0017236. <http://www.ncbi.nlm.nih.gov/pubmed/21359146>.

37. Brandenberger C, Rowley NL, Jackson-Humbles DN, Zhang Q, Bramble LA, Lewandowski RP, et al. Engineered silica nanoparticles act as adjuvants to enhance allergic airway disease in mice. Particle and fibre toxicology. 2013;10:26; doi: 10.1186/1743-8977-10-26. <http://www.ncbi.nlm.nih.gov/pubmed/23815813>.

38. Park HJ, Sohn JH, Kim YJ, Park YH, Han H, Park KH, et al. Acute exposure to silica nanoparticles aggravate airway inflammation: different effects according to surface characteristics. Experimental & molecular medicine. 2015;47:e173; doi: 10.1038/emm.2015.50. <http://www.ncbi.nlm.nih.gov/pubmed/26183169>.

39. Han H, Park YH, Park HJ, Lee K, Um K, Park JW, et al. Toxic and adjuvant effects of silica nanoparticles on ovalbumin-induced allergic airway inflammation in mice. Respiratory research. 2016;17 1:60; doi: 10.1186/s12931-016-0376-x. <http://www.ncbi.nlm.nih.gov/pubmed/27194244>.

40. Rossi EM, Pylkkanen L, Koivisto AJ, Nykasenoja H, Wolff H, Savolainen K, et al. Inhalation exposure to nanosized and fine TiO2 particles inhibits features of allergic asthma in a murine model. Particle and fibre toxicology. 2010;7:35; doi: 10.1186/1743-8977-7-35. <http://www.ncbi.nlm.nih.gov/pubmed/21108815>.

41. Gustafsson A, Jonasson S, Sandstrom T, Lorentzen JC, Bucht A. Genetic variation influences immune responses in sensitive rats following exposure to TiO2 nanoparticles. Toxicology. 2014;326:74-85; doi: 10.1016/j.tox.2014.10.004. <http://www.ncbi.nlm.nih.gov/pubmed/25456268>.

42. Huang KL, Lee YH, Chen HI, Liao HS, Chiang BL, Cheng TJ. Zinc oxide nanoparticles induce eosinophilic airway inflammation in mice. Journal of hazardous materials. 2015;297:304-12; doi: 10.1016/j.jhazmat.2015.05.023. <http://www.ncbi.nlm.nih.gov/pubmed/26010476>.

43. Mizutani N, Nabe T, Yoshino S. Exposure to multiwalled carbon nanotubes and allergen promotes early- and late-phase increases in airway resistance in mice. Biological & pharmaceutical bulletin. 2012;35 12:2133-40. <http://www.ncbi.nlm.nih.gov/pubmed/23207765>.

44. Park EJ, Cho WS, Jeong J, Yi J, Choi K, Park K. Pro-inflammatory and potential allergic responses resulting from B cell activation in mice treated with multi-walled carbon nanotubes by intratracheal instillation. Toxicology. 2009;259 3:113-21; doi: 10.1016/j.tox.2009.02.009. <http://www.ncbi.nlm.nih.gov/pubmed/19428951>.

45. Ryman-Rasmussen JP, Tewksbury EW, Moss OR, Cesta MF, Wong BA, Bonner JC. Inhaled multiwalled carbon nanotubes potentiate airway fibrosis in murine allergic asthma. American journal of respiratory cell and molecular biology. 2009;40 3:349-58; doi: 10.1165/rcmb.2008-0276OC. <http://www.ncbi.nlm.nih.gov/pubmed/18787175>.

46. Thompson EA, Sayers BC, Glista-Baker EE, Shipkowski KA, Ihrie MD, Duke KS, et al. Role of signal transducer and activator of transcription 1 in murine allergen-induced airway remodeling and exacerbation by carbon nanotubes. Am J Respir Cell Mol Biol. 2015;53 5:625-36; doi: 10.1165/rcmb.2014-0221OC. <http://www.ncbi.nlm.nih.gov/pubmed/25807359>.

47. Sayers BC, Taylor AJ, Glista-Baker EE, Shipley-Phillips JK, Dackor RT, Edin ML, et al. Role of cyclooxygenase-2 in exacerbation of allergen-induced airway remodeling by multiwalled carbon nanotubes. Am J Respir Cell Mol Biol. 2013;49 4:525-35; doi: 10.1165/rcmb.2013-0019OC. <http://www.ncbi.nlm.nih.gov/pubmed/23642096>.

48. Ronzani C, Casset A, Pons F. Exposure to multi-walled carbon nanotubes results in aggravation of airway inflammation and remodeling and in increased production of epithelium-derived innate cytokines in a mouse model of asthma. Archives of toxicology. 2014;88 2:489-99; doi: 10.1007/s00204-013-1116-3. <http://www.ncbi.nlm.nih.gov/pubmed/23948970>.

49. Inoue K, Koike E, Yanagisawa R, Hirano S, Nishikawa M, Takano H. Effects of multi-walled carbon nanotubes on a murine allergic airway inflammation model. Toxicology and applied pharmacology. 2009;237 3:306-16; doi: 10.1016/j.taap.2009.04.003. <http://www.ncbi.nlm.nih.gov/pubmed/19371758>.

50. Inoue K, Yanagisawa R, Koike E, Nishikawa M, Takano H. Repeated pulmonary exposure to single-walled carbon nanotubes exacerbates allergic inflammation of the airway: Possible role of oxidative stress. Free radical biology & medicine. 2010;48 7:924-34; doi: 10.1016/j.freeradbiomed.2010.01.013. <http://www.ncbi.nlm.nih.gov/pubmed/20093178>.

51. Lefebvre DE, Pearce B, Fine JH, Chomyshyn E, Ross N, Halappanavar S, et al. In vitro enhancement of mouse T helper 2 cell sensitization to ovalbumin allergen by carbon black nanoparticles. Toxicol Sci. 2014;138 2:322-32; doi: 10.1093/toxsci/kfu010. <http://www.ncbi.nlm.nih.gov/pubmed/24449417>.

52. Ryan JJ, Bateman HR, Stover A, Gomez G, Norton SK, Zhao W, et al. Fullerene nanomaterials inhibit the allergic response. J Immunol. 2007;179 1:665-72. <http://www.ncbi.nlm.nih.gov/pubmed/17579089>.

53. Murr LE, Garza KM, Soto KF, Carrasco A, Powell TG, Ramirez DA, et al. Cytotoxicity assessment of some carbon nanotubes and related carbon nanoparticle aggregates and the implications for anthropogenic carbon nanotube aggregates in the environment. International journal of environmental research and public health. 2005;2 1:31-42. <http://www.ncbi.nlm.nih.gov/pubmed/16705799>.

54. Hussain S, Sangtian S, Anderson SM, Snyder RJ, Marshburn JD, Rice AB, et al. Inflammasome activation in airway epithelial cells after multi-walled carbon nanotube exposure mediates a profibrotic response in lung fibroblasts. Particle and fibre toxicology. 2014;11:28; doi: 10.1186/1743-8977-11-28. <http://www.ncbi.nlm.nih.gov/pubmed/24915862>.

55. Shipkowski KA, Taylor AJ, Thompson EA, Glista-Baker EE, Sayers BC, Messenger ZJ, et al. An Allergic Lung Microenvironment Suppresses Carbon Nanotube-Induced Inflammasome Activation via STAT6-Dependent Inhibition of Caspase-1. PloS one. 2015;10 6:e0128888; doi: 10.1371/journal.pone.0128888. <http://www.ncbi.nlm.nih.gov/pubmed/26091108>.

56. Chen EY, Garnica M, Wang YC, Chen CS, Chin WC. Mucin secretion induced by titanium dioxide nanoparticles. PloS one. 2011;6 1:e16198; doi: 10.1371/journal.pone.0016198. <http://www.ncbi.nlm.nih.gov/pubmed/21283816>.

57. Radauer-Preiml I, Andosch A, Hawranek T, Luetz-Meindl U, Wiederstein M, Horejs-Hoeck J, et al. Nanoparticle-allergen interactions mediate human allergic responses: protein corona characterization and cellular responses. Particle and fibre toxicology. 2016;13:3; doi: 10.1186/s12989-016-0113-0. <http://www.ncbi.nlm.nih.gov/pubmed/26772182>.

58. de Haar C, Kool M, Hassing I, Bol M, Lambrecht BN, Pieters R. Lung dendritic cells are stimulated by ultrafine particles and play a key role in particle adjuvant activity. J Allergy Clin Immunol. 2008;121 5:1246-54; doi: 10.1016/j.jaci.2008.01.010. <http://www.ncbi.nlm.nih.gov/pubmed/18313130>.

59. Chen EY, Garnica M, Wang YC, Mintz AJ, Chen CS, Chin WC. A mixture of anatase and rutile TiO(2) nanoparticles induces histamine secretion in mast cells. Particle and fibre toxicology. 2012;9:2; doi: 10.1186/1743-8977-9-2. <http://www.ncbi.nlm.nih.gov/pubmed/22260553>.

60. Hirai T, Yoshioka Y, Takahashi H, Ichihashi K, Yoshida T, Tochigi S, et al. Amorphous silica nanoparticles enhance cross-presentation in murine dendritic cells. Biochemical and biophysical research communications. 2012;427 3:553-6; doi: 10.1016/j.bbrc.2012.09.095. <http://www.ncbi.nlm.nih.gov/pubmed/23022188>.

61. Chen W, Zhang Q, Kaplan BL, Baker GL, Kaminski NE. Induced T cell cytokine production is enhanced by engineered nanoparticles. Nanotoxicology. 2014;8 Suppl 1:11-23; doi: 10.3109/17435390.2013.848302. <http://www.ncbi.nlm.nih.gov/pubmed/24256152>.

62. Becker S, Soukup J. Coarse(PM(2.5-10)), fine(PM(2.5)), and ultrafine air pollution particles induce/increase immune costimulatory receptors on human blood-derived monocytes but not on alveolar macrophages. Journal of toxicology and environmental health Part A. 2003;66 9:847-59; doi: 10.1080/15287390306381. <http://www.ncbi.nlm.nih.gov/pubmed/12746131>.

63. Nakanishi K, Tsukimoto M, Tanuma S, Takeda K, Kojima S. Silica nanoparticles activate purinergic signaling via P2X7 receptor in dendritic cells, leading to production of pro-inflammatory cytokines. Toxicology in vitro : an international journal published in association with BIBRA. 2016;35:202-11; doi: 10.1016/j.tiv.2016.06.003. <http://www.ncbi.nlm.nih.gov/pubmed/27311643>.

64. Blank F, Gerber P, Rothen-Rutishauser B, Sakulkhu U, Salaklang J, De Peyer K, et al. Biomedical nanoparticles modulate specific CD4+ T cell stimulation by inhibition of antigen processing in dendritic cells. Nanotoxicology. 2011;5 4:606-21; doi: 10.3109/17435390.2010.541293. <http://www.ncbi.nlm.nih.gov/pubmed/21231795>.

65. Winter M, Beer HD, Hornung V, Kramer U, Schins RP, Forster I. Activation of the inflammasome by amorphous silica and TiO2 nanoparticles in murine dendritic cells. Nanotoxicology. 2011;5 3:326-40; doi: 10.3109/17435390.2010.506957. <http://www.ncbi.nlm.nih.gov/pubmed/20846021>.

66. Schanen BC, Das S, Reilly CM, Warren WL, Self WT, Seal S, et al. Immunomodulation and T helper TH(1)/TH(2) response polarization by CeO(2) and TiO(2) nanoparticles. PloS one. 2013;8 5:e62816; doi: 10.1371/journal.pone.0062816. <http://www.ncbi.nlm.nih.gov/pubmed/23667525>.

67. Koike E, Takano H, Inoue K, Yanagisawa R, Kobayashi T. Carbon black nanoparticles promote the maturation and function of mouse bone marrow-derived dendritic cells. Chemosphere. 2008;73 3:371-6; doi: 10.1016/j.chemosphere.2008.05.054. <http://www.ncbi.nlm.nih.gov/pubmed/18602660>.

68. Andersson-Willman B, Gehrmann U, Cansu Z, Buerki-Thurnherr T, Krug HF, Gabrielsson S, et al. Effects of subtoxic concentrations of TiO2 and ZnO nanoparticles on human lymphocytes, dendritic cells and exosome production. Toxicology and applied pharmacology. 2012;264 1:94-103; doi: 10.1016/j.taap.2012.07.021. <http://www.ncbi.nlm.nih.gov/pubmed/22842014>.

69. Villiers C, Freitas H, Couderc R, Villiers MB, Marche P. Analysis of the toxicity of gold nano particles on the immune system: effect on dendritic cell functions. Journal of nanoparticle research : an interdisciplinary forum for nanoscale science and technology. 2010;12 1:55-60; doi: 10.1007/s11051-009-9692-0. <http://www.ncbi.nlm.nih.gov/pubmed/21841911>.

70. Ko JW, Park JW, Shin NR, Kim JH, Cho YK, Shin DH, et al. Copper oxide nanoparticle induces inflammatory response and mucus production via MAPK signaling in human bronchial epithelial cells. Environmental toxicology and pharmacology. 2016;43:21-6; doi: 10.1016/j.etap.2016.02.008. <http://www.ncbi.nlm.nih.gov/pubmed/26934431>.

71. Umemoto EY, Speck M, Shimoda LM, Kahue K, Sung C, Stokes AJ, et al. Single-walled carbon nanotube exposure induces membrane rearrangement and suppression of receptor-mediated signalling pathways in model mast cells. Toxicology letters. 2014;229 1:198-209; doi: 10.1016/j.toxlet.2014.06.009. <http://www.ncbi.nlm.nih.gov/pubmed/24910985>.

72. Aldossari AA, Shannahan JH, Podila R, Brown JM. Influence of physicochemical properties of silver nanoparticles on mast cell activation and degranulation. Toxicology in vitro : an international journal published in association with BIBRA. 2015;29 1:195-203; doi: 10.1016/j.tiv.2014.10.008. <http://www.ncbi.nlm.nih.gov/pubmed/25458489>.

73. Babin K, Antoine F, Goncalves DM, Girard D. TiO2, CeO2 and ZnO nanoparticles and modulation of the degranulation process in human neutrophils. Toxicology letters. 2013;221 1:57-63; doi: 10.1016/j.toxlet.2013.05.010. <http://www.ncbi.nlm.nih.gov/pubmed/23726862>.

74. Kim YM, Reed W, Lenz AG, Jaspers I, Silbajoris R, Nick HS, et al. Ultrafine carbon particles induce interleukin-8 gene transcription and p38 MAPK activation in normal human bronchial epithelial cells. American journal of physiology Lung cellular and molecular physiology. 2005;288 3:L432-41; doi: 10.1152/ajplung.00285.2004. <http://www.ncbi.nlm.nih.gov/pubmed/15695543>.

75. Yamaki K, Yoshino S. Comparison of inhibitory activities of zinc oxide ultrafine and fine particulates on IgE-induced mast cell activation. Biometals : an international journal on the role of metal ions in biology, biochemistry, and medicine. 2009;22 6:1031-40; doi: 10.1007/s10534-009-9254-z. <http://www.ncbi.nlm.nih.gov/pubmed/19609684>.

76. Johnston H, Brown DM, Kanase N, Euston M, Gaiser BK, Robb CT, et al. Mechanism of neutrophil activation and toxicity elicited by engineered nanomaterials. Toxicology in vitro : an international journal published in association with BIBRA. 2015;29 5:1172-84; doi: 10.1016/j.tiv.2015.04.021. <http://www.ncbi.nlm.nih.gov/pubmed/25962642>.

77. Val S, Hussain S, Boland S, Hamel R, Baeza-Squiban A, Marano F. Carbon black and titanium dioxide nanoparticles induce pro-inflammatory responses in bronchial epithelial cells: need for multiparametric evaluation due to adsorption artifacts. Inhalation toxicology. 2009;21 Suppl 1:115-22; doi: 10.1080/08958370902942533. <http://www.ncbi.nlm.nih.gov/pubmed/19558243>.

78. Ursini CL, Maiello R, Ciervo A, Fresegna AM, Buresti G, Superti F, et al. Evaluation of uptake, cytotoxicity and inflammatory effects in respiratory cells exposed to pristine and -OH and -COOH functionalized multi-wall carbon nanotubes. Journal of applied toxicology : JAT. 2016;36 3:394-403; doi: 10.1002/jat.3228. <http://www.ncbi.nlm.nih.gov/pubmed/26370214>.

79. Frieke Kuper C, Grollers-Mulderij M, Maarschalkerweerd T, Meulendijks NM, Reus A, van Acker F, et al. Toxicity assessment of aggregated/agglomerated cerium oxide nanoparticles in an in vitro 3D airway model: the influence of mucociliary clearance. Toxicology in vitro : an international journal published in association with BIBRA. 2015;29 2:389-97; doi: 10.1016/j.tiv.2014.10.017. <http://www.ncbi.nlm.nih.gov/pubmed/25448805>.

80. Skuland T, Ovrevik J, Lag M, Schwarze P, Refsnes M. Silica nanoparticles induce cytokine responses in lung epithelial cells through activation of a p38/TACE/TGF-alpha/EGFR-pathway and NF-kappaBeta signalling. Toxicology and applied pharmacology. 2014;279 1:76-86; doi: 10.1016/j.taap.2014.05.006. <http://www.ncbi.nlm.nih.gov/pubmed/24844442>.

81. Verstraelen S, Remy S, Casals E, De Boever P, Witters H, Gatti A, et al. Gene expression profiles reveal distinct immunological responses of cobalt and cerium dioxide nanoparticles in two in vitro lung epithelial cell models. Toxicology letters. 2014;228 3:157-69; doi: 10.1016/j.toxlet.2014.05.006. <http://www.ncbi.nlm.nih.gov/pubmed/24821434>.

82. Jing X, Park JH, Peters TM, Thorne PS. Toxicity of copper oxide nanoparticles in lung epithelial cells exposed at the air-liquid interface compared with in vivo assessment. Toxicology in vitro : an international journal published in association with BIBRA. 2015;29 3:502-11; doi: 10.1016/j.tiv.2014.12.023. <http://www.ncbi.nlm.nih.gov/pubmed/25575782>.

83. Park EJ, Zahari NE, Lee EW, Song J, Lee JH, Cho MH, et al. SWCNTs induced autophagic cell death in human bronchial epithelial cells. Toxicology in vitro : an international journal published in association with BIBRA. 2014;28 3:442-50; doi: 10.1016/j.tiv.2013.12.012. <http://www.ncbi.nlm.nih.gov/pubmed/24389112>.

84. Gualtieri M, Skuland T, Iversen TG, Lag M, Schwarze P, Bilanicova D, et al. Importance of agglomeration state and exposure conditions for uptake and pro-inflammatory responses to amorphous silica nanoparticles in bronchial epithelial cells. Nanotoxicology. 2012;6 7:700-12; doi: 10.3109/17435390.2011.604441. <http://www.ncbi.nlm.nih.gov/pubmed/21793771>.

85. Wu W, Samet JM, Peden DB, Bromberg PA. Phosphorylation of p65 is required for zinc oxide nanoparticle-induced interleukin 8 expression in human bronchial epithelial cells. Environmental health perspectives. 2010;118 7:982-7; doi: 10.1289/ehp.0901635. <http://www.ncbi.nlm.nih.gov/pubmed/20194077>.

86. Alazzam A, Mfoumou E, Stiharu I, Kassab A, Darnel A, Yasmeen A, et al. Identification of deregulated genes by single wall carbon-nanotubes in human normal bronchial epithelial cells. Nanomedicine : nanotechnology, biology, and medicine. 2010;6 4:563-9; doi: 10.1016/j.nano.2009.12.005. <http://www.ncbi.nlm.nih.gov/pubmed/20060075>.

87. Hussain S, Boland S, Baeza-Squiban A, Hamel R, Thomassen LC, Martens JA, et al. Oxidative stress and proinflammatory effects of carbon black and titanium dioxide nanoparticles: role of particle surface area and internalized amount. Toxicology. 2009;260 1-3:142-9; doi: 10.1016/j.tox.2009.04.001. <http://www.ncbi.nlm.nih.gov/pubmed/19464580>.
